# Ultrasmall Nanodots with Dual Anti‐Ferropototic Effect for Acute Kidney Injury Therapy

**DOI:** 10.1002/advs.202403305

**Published:** 2024-08-19

**Authors:** Fantian Zeng, Yatong Qin, Sureya Nijiati, Yangtengyu Liu, Jinmin Ye, Huaxiang Shen, Jiayuan Cai, Hehe Xiong, Changrong Shi, Longguang Tang, Chunyang Yu, Zijian Zhou

**Affiliations:** ^1^ State Key Laboratory of Vaccines for Infectious Diseases Xiang An Biomedicine Laboratory School of Public Health Shenzhen Research Institute of Xiamen University Xiamen University Xiamen 361102 China; ^2^ Department of Rheumatology and Immunology Xiangya Hospital Central South University Changsha 410008 China; ^3^ Departments of Diagnostic Radiology, Surgery Chemical and Biomolecular Engineering and Biomedical Engineering Yong Loo Lin School of Medicine and College of Design and Engineering National University of Singapore Singapore 119074 Singapore; ^4^ Gaozhou People's Hospital Maoming 525200 China; ^5^ School of Chemistry and Chemical Engineering State Key Laboratory of Metal Matrix Composites Shanghai Jiao Tong University 800 Dongchuan Road Shanghai 200240 China

**Keywords:** acute kidney injury, ferroptosis, nanodots

## Abstract

Ferroptosis is known to mediate the pathogenesis of chemotherapeutic drug‐induced acute kidney injury (AKI); however, leveraging the benefits of ferroptosis‐based treatments for nephroprotection remains challenging. Here, ultrasmall nanodots, denoted as FerroD, comprising the amphiphilic conjugate (tetraphenylethylene‐_L_‐serine‐deferoxamine, TPE‐lys‐Ser‐DFO (TSD)) and entrapped ferrostatin‐1 are designed. After being internalized through kidney injury molecule‐1‐mediated endocytosis, FerroD can simultaneously remove the overloaded iron ions and eliminate the overproduction of lipid peroxides by the coordination‐disassembly mechanisms, which collectively confer prominent inhibition efficiency of ferroptosis. In cisplatin (CDDP)‐induced AKI mice, FerroD equipped with dual anti‐ferroptotic ability can provide long‐term nephroprotective effects. This study may shed new light on the design and clinical translation of therapeutics targeting ferroptosis for various ferroptosis‐related kidney diseases.

## Graphical abstract

An ultrasmall nanodot (denoted as FerroD) that can simultaneously remove overloaded iron ions and eliminate massive lipid peroxides is reported, in which the dual anti‐ferroptotic ability provided long‐term nephroprotective effect in the kidneys of mice with cisplatin exposure.

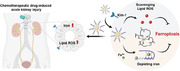

## 1 Introduction

**Figure 1:**
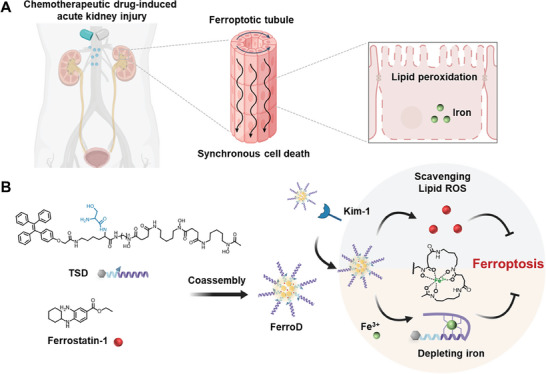
Illustration of ultrasmall nanodots with dual anti‐ferropototic effect for acute kidney injury therapy, denoted as FerroD. A) In the context of chemotherapeutic drug‐induced acute kidney injury, ferroptosis could induce synchronized renal tubule cell death following the intercellular propagation of cell death in a wavelike pattern. Ferroptotic stress in the proximal tubules increases intracellular lipid peroxidation and iron accumulation. B) Amphiphilic conjugate (tetraphenylethylene‐_L_‐serine‐deferoxamine, TSD) could encapsulate ferrostatin‐1 to obtain FerroD, which could be internalized through kidney injury molecule‐1‐mediated endocytosis to simultaneously remove overloaded iron ions and eliminate massive lipid peroxides for the synergistic inhibition of ferroptosis.

Patients receiving chemotherapeutics are often at high risk of acute kidney injury (AKI) due to the inevitable renal excretion of chemotherapy drugs.^[^ ^1^ ^]^ The chemotherapeutic drug‐induced AKI featuring rapid deterioration of renal functions may progress into advanced complications, including chronic kidney disease, renal failure, and death.^[^ ^2^ ^]^ Generally, the pathogenesis of AKI involves diverse modalities of regulated cell death, including ferroptosis, necroptosis, and pyroptosis.^[^ ^3^ ^]^ Among them, ferroptosis, a form of cell death driven by iron‐dependent lipid peroxidation, has been demonstrated to initiate the necrosis of renal tubules in a wavelike manner through the release of damage‐associated molecular patterns (DAMPs).^[^ ^4^ ^]^ Synchronized ferroptotic renal cells subsequently provoke a second wave of inflammatory cytokine‐mediated regulated necrosis, such as necroptosis and pyroptosis, that amplifies kidney dysfunction (**Figure** **1**A).^[^ ^5^ ^]^ In this regard, therapeutic approaches targeting ferroptosis were proposed to diminish the initial wave of cell death for better management of AKI.^[^ ^3d^ ^]^ Recent studies demonstrated that the prophylactic administration of ferroptosis inhibitors with a single dose could effectively mitigate AKI, whereas targeting the secondary necrosis in the context of AKI required multiple doses.^[^ ^3^, ^6^ ^]^ Furthermore, our group previously developed an artemisinin‐based probe (Art–Gd) for contrast‐enhanced magnetic resonance imaging of ferroptosis (feMRI), which provided the imaging evidence for ferroptosis involved in the early stage of AKI.^[^ ^7^ ^]^ Taken together, realizing ferroptosis as a mechanism of the target could benefit from early diagnostic imaging and therapeutic intervention in AKI.

Renal cells undergo ferroptosis triggered by the discoordination of the regulatory redox mechanisms, culminating in massive peroxidation of polyunsaturated phospholipids and labile iron ions.^[^ ^8^ ^]^ The scavenging of peroxyl radicals and the chelation of excess iron could effectively reduce the loss of kidney epithelial cells and recover dysfunctional kidneys.^[^ ^9^ ^]^ Radical‐trapping antioxidant ferrostatin‐1 (Fer‐1) and iron chelator deferoxamine (DFO) have been widely used as ferroptosis inhibitors in kidney diseases and have been demonstrated to provide nephroprotective effects in various models of drug‐induced AKI, such as cisplatin (CDDP).^[^ ^10^ ^]^ and folic‐acid‐induced AKI.^[^ ^11^ ^]^ However, the anti‐ferroptotic effect of DFO is largely compromised due to unfavorable pharmacokinetics and nonspecific biodistribution in vivo.^[^ ^12^ ^]^ Likewise, the instability and the poor solubility of Fer‐1 in the plasma largely restrain its capacity to inhibit ferroptosis in the context of AKI.^[^ ^13^ ^]^ Nanomedicine offers promising alternatives to traditional pharmaceuticals due to their unique physicochemical properties.^[^ ^14^ ^]^ Through integrating the kidney‐targeted moieties (i.e., the renal tubule‐targeted modifier _L_‐serine) and imaging moieties, such as positron emission tomography (PET) and magnetic resonance imaging (MRI), kidney damage in a longitudinal manner could be monitored to benefit the treatment program in AKI.^[^ ^15^ ^]^ Leveraging the merits of nanomaterials, the past decade has witnessed tremendous progress in developing ferroptosis‐based nanomedicine for AKI.^[^ ^16^ ^]^ However, the nephroprotection efficacy is often poor due to the singular ferroptosis inhibition mechanism, which either involves the scavenging of lipid peroxidation or the chelation of iron ions. Therefore, the development of nanoplatforms equipped with multiple ferroptosis inhibition mechanisms is recognized as a highly efficient strategy to improve therapeutic outcomes owing to the potential synergy between different mechanisms.

In this work, we reported ultrasmall nanodots denoted as FerroD, comprising the amphiphilic conjugate (tetraphenylethylene‐_L_‐serine‐deferoxamine, TPE‐lys‐Ser‐DFO (TSD)) and entrapped Fer‐1 for the treatment of AKI (Figure 1B). TSD could be assembled into nanoparticles and encapsulated with Fer‐1 to obtain FerroD. By tuning the weight ratios between TSD and Fer‐1, we attained ultrasmall FerroD nanodots that could cross through the renal filtration threshold. Moreover, since kidney injury molecule‐1 (Kim‐1) is a specifically upregulated protein at the early stage of AKI, the _L_‐serine‐modified FerroD nanodots could leverage the interaction between Kim‐1 and serine to facilitate cellular internalization. Interestingly, after chelating with Fe(III), the FerroD was subjected to disassembly and released the entrapped Fer‐1 through the coordination‐disassembly mechanisms. Utilizing this release mechanism, FerroD could exert simultaneously lipid peroxidation‐scavenging and iron‐chelation abilities to counteract renal ferroptotic cell death, thereby improving the nephroprotective effect. Furthermore, we used a previously developed ferroptosis‐targeted MRI probe, Art–Gd, to follow up on the long‐term anti‐ferroptotic effect of FerroD on CDDP‐induced AKI mice. Taken together, this work aims to clarify that the rational design of nanomedicine for combination therapy targeting ferroptosis mechanisms represents an effective way to alleviate drug‐induced AKI.

## 2 Results

### 2.1 Design and Characterization of FerroD

**Figure 2:**
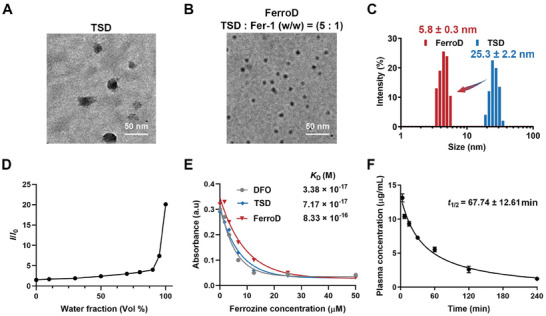
Characterizations of the FerroD. A,B) TEM images of TSD and FerroD. The FerroD was obtained by co‐assembly of TSD and Fer‐1 with a weight ratio (w/w) of 5:1. C) The DLS measurements of TSD (blue) and FerroD (red) samples in saline were repeated three times each. D) Plots of relative emission intensity (*I*/*I*_0_) versus the composition of the aqueous mixtures of TSD. E) UV absorbance curves of the competitive iron binding assay as the function of ferrozine concentration. F) Plasma concentration of TSD molecules on FerroD at different time points. The concentration of TSD in plasma at each time point was calculated from fluorescence signal intensity.

The amphiphilic conjugate TSD was synthesized by chemically conjugating hydrophobic TPE with _L_‐serine and hydrophilic DFO via a lysine linker. The intermediates and the final products were characterized by proton nuclear magnetic resonance (^1^H NMR) spectrum and mass spectrometry (MS) (Figures S1–S9, Supporting Information). The amphiphilic property of TSD led to the self‐assembly into particles with a hydrodynamic diameter of 25.3 ± 2.2 nm, as revealed by the transmission electron microscopy (TEM) image and dynamic light scattering (DLS) measurements (**Figure** **2**A–C). We performed the co‐assembly study of TSD with Fer‐1 using a typical solvent‐evaporation method. By adjusting the weight ratio (w/w) of TSD:Fer‐1, we obtained an ultrasmall nanodot FerroD with a hydrodynamic diameter of 5.8 ± 0.3 nm, which could cross through the renal filtration threshold.^[^ ^17^ ^]^ As a control, the hydrodynamic diameter of Fer‐1@TD without _L_‐serine moiety was 8.3 ± 1.1 nm (Figure S10, Supporting Information). In addition, the DLS result demonstrated that the diameter of FerroD and Fer‐1@TD had negligible changes in phosphate‐buffered saline (PBS) and fetal bovine serum (FBS) solutions for 120 h, indicating their high stability in a biological environment (Figure S11, Supporting Information). The drug‐loading content (DLC) of Fer‐1 in FerroD was found to be 11.19 ± 1.38 wt% (weight percentage). Of note, we observed that TSD exhibited the aggregation‐induced emission (AIE) effect during the self‐assembly process, which is attributed to the restricted intramolecular rotation of the phenyl rings of the TSD molecules. As shown in Figure 2D, TSD molecules dissolved in tetrahydrofuran (THF) showed weak fluorescence, yet the fluorescence intensity increased dramatically when the fraction of H_2_O to THF in solution reached 90%. Then we determined the iron‐binding affinity of FerroD, TSD, and DFO by using the ferrozine competition assay. The dissociation constant (*K*_D_) of TSD was comparable to that of DFO, 7.17 × 10^−17^ versus 3.38 × 10^−17^, indicating that the TSD could effectively chelate iron ions (Figure 2E). The *K*_D_ of FerroD was slightly increased to 8.33 × 10^−16^, which could be due to the increased intramolecular steric effect of DFO moieties on FerroD. The pharmacokinetics study showed that the half‐life (*t*_1/2_ = 67.7 ± 12.61 min) of the TSD molecules on FerroD was substantially prolonged compared with that of DFO alone (*t*_1/2_ = 5–15 min) in the bloodstream of mice (Figure 2F).^[^ ^18^ ^]^

**Figure 3:**
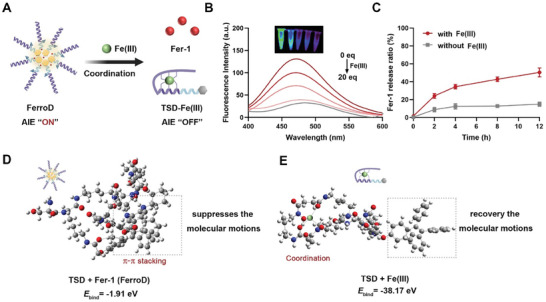
The release mechanism of Fer‐1 from the FerroD. A) Schematic illustration of the coordination‐disassembly mechanisms. B) Fluorescence spectra (*λ*_ex_ = 355 nm) of FerroD upon titration of Fe(III). Inset: Fluorescence photographs of FerroD at different concentrations of Fe(III) from 0 to 20 equivalents. The fluorescence excitation was set at 465 nm. C) The Fer‐1 release profiles of FerroD in the presence or absence of Fe(III) monitored by HPLC. D,E) DFT calculation for the TSD + Fer‐1 (FerroD) and TSD + Fe(III).

Next, we investigated the release profiles of Fer‐1 from FerroD. We speculated that the linear DFO on the TSD could be transformed into a stable hexadentate complex with an octahedral architecture after coordination with Fe(III),^[^ ^19^ ^]^ resulting in the disassembly of FerroD and subsequently the release of Fer‐1 molecules (**Figure** **3**A). To confirm this hypothesis, we measured the fluorescence spectrum of FerroD in the presence of different concentrations of Fe(III). As shown in Figure 3B, FerroD showed a strong emission spectrum at around 465 nm due to the AIE effect in the absence of Fe(III). However, with the increase of Fe(III), the fluorescence intensity at 465 nm decreased, indicating the recovery of intramolecular motion of TPE motifs. In this regard, increasing the rotation of phenyl rings in TPE may enhance the molecular perturbation, leading to the release of the encapsulated Fer‐1. We then quantified the cumulative release of Fer‐1 from FerroD in PBS with or without Fe(III) at different time points through high‐performance liquid chromatography (HPLC) measurement. FerroD was relatively stable in PBS with about 10% release of Fer‐1 within 12 h (Figure 3C). However, in the presence of Fe(III), over 55% of Fer‐1 was released, indicating the disintegration of FerroD. To further understand the coordination‐disassembly mechanism, we carried out density functional theory (DFT) calculations. The binding energy of the TSD to Fe(III) was calculated as −38.17 eV, which was around 20‐fold lower than that of the TSD to Fer‐1 (−1.91 eV) (Figure 3D,E). As a result, the stability of the TSD‐Fe(III) complex is significantly higher than that of the TSD‐Fer‐1 complex (FerroD). In this context, when adding Fe(III), FerroD may undergo a structural transformation to achieve a stable state of the system, resulting in the release of Fer‐1 with weak binding energy. Furthermore, the conformational analysis showed that the rotation freedom of the TPE moiety in the TSD‐Fe(III) complex was higher than that in the TSD‐Fer‐1 complex, which may lead to suppression of the AIE effect. These results were consistent with the conclusions derived from the fluorescence assay.

### 2.2 The Anti‐Ferroptotic Activity of FerroD In Vitro

**Figure 4:**
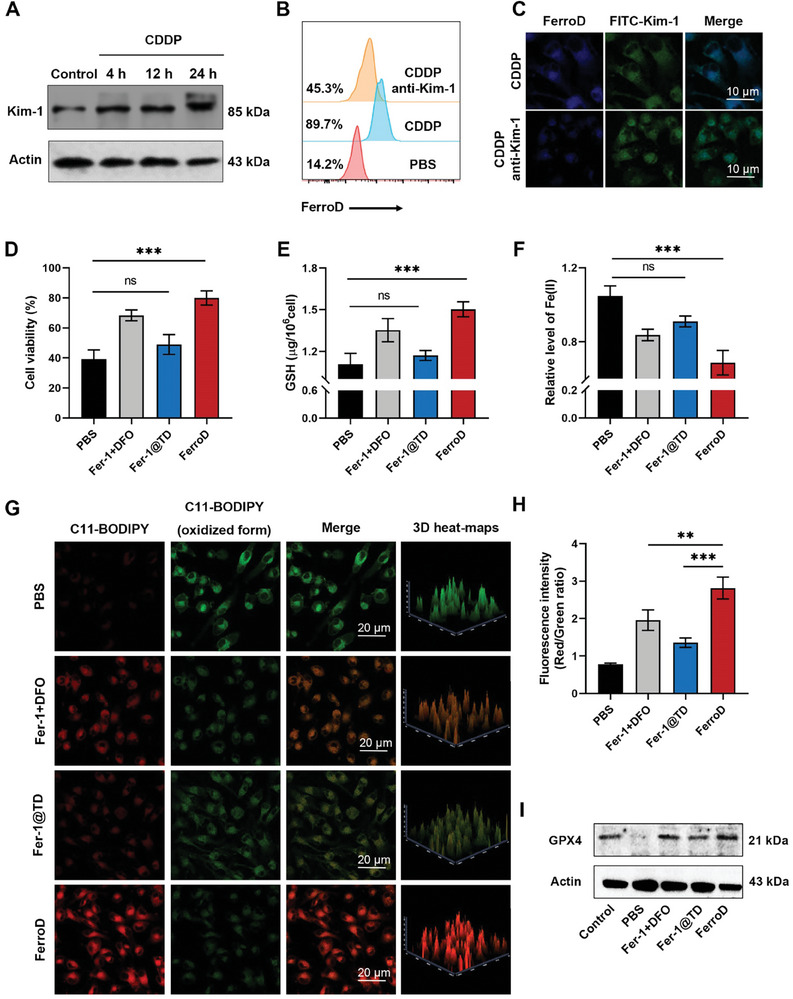
The anti‐ferroptotic activity of FerroD on CDDP‐induced HK‐2 cells in vitro. A) Western blotting analysis of Kim‐1 expression using lysates from normal and CDDP‐induced HK‐2 cells at different time points. B) The flow cytometry analysis of the cellular uptake of the FerroD in the PBS‐treated HK‐2 cells and CDDP‐induced HK‐2 cells pretreated with and without the Kim‐1 antibody. C) Representative confocal images of colocalization of FerroD and Kim‐1 in CDDP‐induced HK‐2 cells pretreated with and without the Kim‐1 antibody. FerroD is shown in blue. Green indicates fluorescein isothiocyanate (FITC)‐labeled Kim‐1 antibody. scale bar = 10 µm. D–F) The cell viability profiles, GSH, and Fe(II) of CDDP‐induced HK‐2 cells after different treatments, including saline, Fer‐1 + DFO, Fer‐1@TD, and FerroD (*n* = 3 per group, ^***^ *P* < 0.001). G,H) The confocal fluorescence images and the quantitative analysis of fluorescence intensity (Red/Green ratio) of CDDP‐induced HK‐2 cells after different treatments (^**^ *P* < 0.01, ^***^ *P* < 0.001), scale bar = 20 µm. I) Western blotting analysis of GPX4 expression in CDDP‐induced HK‐2 cells after different treatments.

We first performed 3‐(4,5‐dimethylthiazol‐2‐yl)−2,5‐diphenyltetrazolium bromide (MTT) assays to study the cytotoxicity of the FerroD toward human kidney 2 (HK‐2) cells. The results showed that the FerroD probe has negligible cytotoxicity at concentrations up to 200 µm (for TSD), indicating the good biocompatibility of the FerroD (Figure S12, Supporting Information). Next, the cellular internalization of FerroD by HK‐2 cells treated with CDDP was investigated. We first evaluated the levels of Kim‐1 in CDDP‐treated HK‐2 cells at different time points by western blotting (WB) assays. The results showed that the expression of Kim‐1 significantly increased at 4 h after CDDP treatment (**Figure** **4**A; Figure S13, Supporting Information). The cellular uptake of FerroD in CDDP‐treated HK‐2 cells was then studied by flow cytometry. As shown in Figure 4B, the CDDP‐treated HK‐2 cells showed an obviously enhanced fluorescence signal compared with that of untreated HK‐2 cells. After being treated with the anti‐Kim‐1 antibody, the fluorescence signal of the CDDP‐treated HK‐2 cells greatly diminished, further confirming the Kim‐1‐specific targeting ability of FerroD. As a control, Fer‐1@TD without the Kim‐1‐targeting moiety exhibited no significant fluorescence change in both CDDP‐treated and untreated HK‐2 cells (Figure S14, Supporting Information). Furthermore, we observed that the CDDP‐treated HK‐2 cells incubated with the FerroD showed strong colocalized signals with the fluorescein isothiocyanate (FITC)‐labeled Kim‐1 antibody by confocal laser scanning microscopy (CLSM) (Figure 4C). These results demonstrated that the upregulation of Kim‐1 in CDDP‐treated HK‐2 cells could facilitate the cellular uptake of the FerroD.

Given that CDDP nephrotoxicity was involved in various ferroptotic stresses in the proximal tubules, such as overproduced lipid peroxides (LPO) and overloaded iron ions,^[^ ^20^ ^]^ we investigated the cytoprotection of FerroD in CDDP‐treated HK‐2 cells. As shown in Figure 4D, the cell viability of CDDP‐treated HK‐2 cells improved from 47.6% to 81.5% through the treatment of FerroD even at a concentration of 2.5 µg mL^−1^ (Figure S15A, Supporting Information). Under the same conditions, the cell viability of the single‐drug treated group (Fer‐1 and DFO) and Fer‐1@TD‐treated group did not significantly change compared to that of the PBS group (Figure S15B, Supporting Information). It is noteworthy that the cell viability of HK‐2 cells treated with the Fer‐1 + DFO group slightly decreased compared with that of FerroD, which may be ascribed to the varied cellular uptake efficiency and the delivery kinetics of DFO and Fer‐1. In addition, we also evaluated the cytoprotection of FerroD in erastin (ferroptosis inducer)‐treated HK‐2 cells. As expected, FerroD substantially augmented the cell viability of erastin‐treated HK‐2 cells, from 33.1% to 88.2% (Figure S16, Supporting Information). To further understand the molecular mechanism for the cytoprotective effect of the FerroD, we evaluated the intracellular ferrous ions and the antioxidant glutathione (GSH) level using commercial kits. As expected, FerroD could increase the GSH level and reduce the intracellular ferrous ions (Figure 4E,F). On the one hand, TSD could chelate the overloaded iron ions to reduce reactive oxygen species (ROS) generation mediated by the Fenton reaction. On the other hand, the release of Fer‐1 with strong radical‐trapping activity could greatly improve the antioxidant‐buffering capability intracellularly. To validate the cellular antioxidant protective effect of FerroD, we further performed CLSM imaging of C11‐BODIPY^581/591^, an LPO‐specific fluorescent probe, to assess the accumulation of lipid ROS. Upon C11‐BODIPY^581/591^ oxidization, the maximum emission peak shifted from 590 nm (red) to 510 nm (green). As revealed in Figure 4G, FerroD‐treated HK‐2 cells showed much stronger red fluorescence than that of the Fer‐1 + DFO and Fer‐1@TD groups. The quantitative analysis demonstrated that the fluorescence intensity (red/green ratio) of the FerroD group was 1.44‐ and 2.15‐folds higher than that of the Fer‐1 + DFO group and Fer‐1@TD group, respectively, indicating the prominent anti‐ferroptotic ability of the FerroD (Figure 4H). Furthermore, we investigated the expression of glutathione peroxidase 4 (GPX4), which is an antioxidative enzyme that could reduce the level of LPO. WB results showed that FerroD‐treated HK‐2 cells significantly upregulated GPX4 expression after CDDP treatment (Figure 4I; Figure S17, Supporting Information). Collectively, the above results revealed that FerroD could alleviate CDDP‐treated cell death in vitro through its robust anti‐ferroptotic effect.

### 2.3 Renal Clearance Efficiency and Biodistribution of FerroD

**Figure 5:**
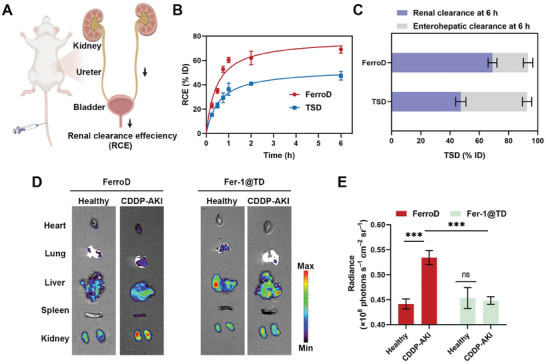
Renal clearance efficiency and biodistribution of FerroD. A) Schematic illustration of the excretion of FerroD through the urinary tract. B) RCE of FerroD and TSD at different post‐injection times. C) The amount of TSD excreted from the renal pathway (purple) and enterohepatic pathway (gray) of mice at 6 h post‐injection. D) Fluorescence images of the main organs (heart, lung, liver, spleen, and kidney) of the normal and AKI mice at 1 h after intravenous injection of FerroD or Fer‐1@TD. E) The quantitation of the fluorescence intensity of kidneys of the normal and AKI mice at 1 h after intravenous injection of FerroD or Fer‐1@TD.

Prior to investigating the therapeutic efficacy of the FerroD against AKI in vivo, we first evaluated the biocompatibility of FerroD. Hematoxylin and eosin (H&E) staining images of major organs after intravenous (i.v.) injection of FerroD showed little to no abnormality in tissues, indicating negligible systemic toxicity (Figure S18, Supporting Information). Furthermore, as shown in Figures S19 and S20 (Supporting Information), no significant changes in blood biochemistry and hematology were observed in saline‐ and FerroD‐treated mice. Subsequently, we determined the renal clearance efficiency (RCE) of FerroD by quantifying the fluorescence intensity of urine at different times after intravenous injection (**Figure** **5**A). The results demonstrated that the RCE of FerroD was higher than that of TSD (52.60% vs 36.33% ID) at 1 h post injection, which may be attributed to the smaller size of FerroD (Figure 5B). As a control, the RCE of Fer‐1@TD was 40.60% ID at 1 h post injection (Figure S21, Supporting Information). Moreover, we collected and homogenized the major organs of the mice to analyze the distribution of residuals in the body. As shown in Figure 5C, the proportion of enterohepatic clearance for FerroD was lower than that of TSD. We then established an AKI mouse model by intraperitoneal (i.p.) injection of CDDP (15 mg kg^−1^) and investigated the biodistribution of FerroD in AKI mice. Fluorescence images of major organs obtained from normal and AKI mice were captured at 1 h after intravenous injection of FerroD (Figure 5D,E; Figure S22, Supporting Information). The fluorescent signals of FerroD were markedly higher in the kidneys of AKI mice than in normal mice, indicating that FerroD could be rapidly cleared by the normal kidney but highly uptake by the AKI kidney. For Fer‐1@TD, the fluorescent signals in the kidneys of normal and AKI mice were unobviously different. The nonspecific uptake of FerroD in the liver was possibly due to serum protein adsorption and subsequent uptake by macrophages.^[^ ^21^ ^]^

### 2.4 The Therapeutic Efficacy of FerroD in CDDP‐Induced AKI Mice

**Figure 6:**
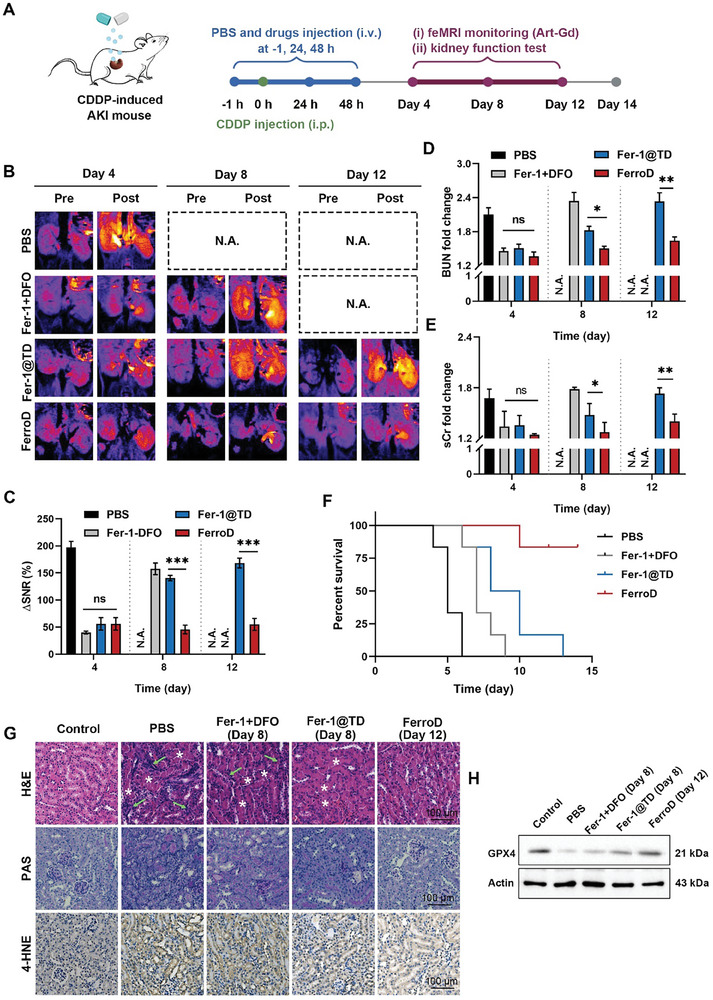
The therapeutic efficacy of FerroD on CDDP‐induced AKI mice. A) Scheme shows the experimental procedure. Mice were treated with an intraperitoneal injection of CDDP (15 mg kg^−1^). Therapeutic drugs were administered 1 h before CDDP injection and 24 and 48 h after CDDP injection. *T*_1_‐weighted MRI was conducted to acquire pre‐ and postcontrast *T*_1_ MRI before and after intravenous injection of the Art–Gd probe (1.12 × 10^3^ µm Gd) on days 4, 8, and 12 after treatment with CDDP. B) Representative *T*_1_‐weighted images (pseudocolor) of mouse kidneys from different groups at pre‐ and post‐contrast points were acquired on days 4, 8, and 12 after treatment with CDDP. N.A. means not available due to the death of the mouse group. C) Quantification of ΔSNR of the parenchyma for the *T*_1_‐weighted images from different groups (*n* = 4 per group; ns represents no significance, ^**^ *P* < 0.01; ^***^ *P* < 0.001). D,E) The BUN and sCr levels of mice after different treatments on days 4, 8, and 12 after treatment with CDDP (^*^ *P* < 0.05; ^**^ *P* < 0.01). F) The mouse survival rates of AKI mice were recorded for 14 days after different treatments. G) Representative images of H&E, PAS, and 4‐HNE staining of kidney tissues after different treatments. Asterisks denoted cast formation; green arrows denoted inflammatory cell infiltration, scale bar = 100 µm. H) Western blotting analysis of GPX4 expression in kidney tissues of CDDP‐induced AKI mice before treatment with different treatments.

Encouraged by the anti‐ferroptotic properties in vitro and the favorable renal accumulation features in vivo, we further investigated the therapeutic effect of FerroD for alleviating CDDP‐induced AKI in a mouse model (**Figure** **6**A). Mice were randomly divided into five groups (*n* = 4) with different treatments: healthy mice + saline, AKI mice + saline, AKI mice + Fer‐1 + DFO, AKI mice + Fer‐1@TD, and AKI mice + FerroD. The anti‐ferroptosis ability of different groups was assessed in real‐time using the Art–Gd probe, which is designed to feMRI targeting the reactivity of Fe(II). The radical production of the Art–Gd probe in the presence of Fe(II) leads to the formation of Art–Gd–protein complexes. The slow tumbling feature of the obtained complexes would enhance longitudinal relaxation time (*T*_1_) contrast.^[^ ^7^ ^]^ We performed *T*_1_‐weighted MRI of the kidney for different groups and subsequently obtained the multislice *T*_1_‐weighted images before (pre‐) and after (post‐) the administration of the Art–Gd probe (Figures S23–S26, Supporting Information). The representative *T*_1_‐weighted images at pre‐ and post contrast of a coronal slice of the kidney from each group are shown in Figure 6B. On day 4, after treatment with CDDP, the kidneys of saline‐treated mice showed a stronger *T*_1_ bright contrast compared with those of healthy mice, which was attributed to the Fe(II) accumulation caused by CDDP‐triggered ferroptosis. The semiquantitative signal‐to‐noise ratio (SNR) analysis indicated that the ΔSNR_kidney_ = [(SNR_pre_ − SNR_post_)/SNR_pre_] of CDDP‐induced AKI mice was 3.5‐fold higher than that of normal mice on day 4, 195.4 ± 2.6% versus 55.0 ± 3.7%, yet negligible contrast change was observed in the Fer‐1 + DFO, Fer‐1@TD, and FerroD treatment groups with ΔSNR_kidney_ values of 42.0 ± 2.6%, 56.0 ± 11.3%, and 51.3 ± 13.9%, respectively (Figure 6C). Furthermore, we found that the ΔSNR_kidney_ of mice treated with the Fer‐1 + DFO and Fer‐1@TD showed a significant increase on day 8, implying that these two treatments failed to impede the progression of AKI mice due to their limited anti‐ferroptotic ability. Of note, the kidneys of the FerroD‐treated mice maintained weak contrast enhancement on day 12 after treatment with CDDP, which suggested that the FerroD could act as a potent ferroptosis inhibitor to protect kidneys from the nephrotoxicity of CDDP in a long term. To further verify the therapeutic effect of different treatments, we determined the biomarkers representing kidney functions, including serum creatinine (sCr), blood urea nitrogen (BUN), Kim‐1, and neutrophil gelatinase‐associated lipocalin (NGAL) at different time points after treatment with CDDP (Figure 6D,E). The sCr and BUN levels in the saline treatment groups exhibited an ≈1.8‐fold increase compared to those of healthy levels on day 4, while those that received the Fer‐1 + DFO and Fer‐1@TD treatments reached similar levels on day 8. It should be noted that the sCr and BUN levels of the FerroD‐treated mice were within the normal range during the treatment period of 12 days. The changes in the Kim‐1 and NGAL levels of the different treatment groups were consistent with those in the sCr levels (Figure S27, Supporting Information). In addition, FerroD‐treated AKI mice hold a survival time of more than 14 days longer than those of other treatment groups (Figure 6F). To evaluate the histopathological damage of AKI, we performed H&E and periodic acid Schiff (PAS) staining. As shown in Figure 6G, the hyaline cast formation and inflammatory cell infiltration could be clearly observed in the kidney sections in the treatment groups with Fer‐1 + DFO and Fer‐1@TD on day 8 post treatment, while much fewer damaged structures could be found in the treatment group with FerroD on day 12 post treatment. Furthermore, we also conducted 4‐hydroxynonenal (4‐HNE) staining and WB assays for the evaluation of LPO accumulation and GPX4 levels, respectively. The results showed that the kidney tissues of FerroD‐treated mice had a lower proportion of 4‐HNE‐positive ratio and more GPX4 expression than those of other treatment groups (Figure 6H; Figure S28, Supporting Information). Additionally, the total iron content in the kidneys of mice treated with FerroD was comparable to that in normal kidneys (Figure S29, Supporting Information). Taken together, the above results demonstrated that the FerroD was efficient in exerting a nephroprotective effect through dual anti‐ferroptotic ability.

## 3 Discussion

It is well known that chemotherapeutic drug‐induced AKI often leads to dose reduction or cessation of the treatment.^[^ ^22^ ^]^ Understanding the underlying molecular mechanisms of renal cells under chemical stressors could benefit the deployment of AKI. Mounting evidence indicated that the initiation of AKI involved the execution and regulation of multiple forms of regulated cell death, which caused kidney injury directly or through the recruitment of immune cells and stimulation of the inflammatory responses.^[^ ^23^ ^]^ Of note, the predominant form of cell death may evolve over time, and different molecular targets may be relevant for the prevention or treatment of AKI.^[^ ^3d^ ^]^ Although pan‐cell death inhibitors that act on two or three pathways may provide some therapeutic benefit, interventions that effectively inhibit the main modality of regulated necrosis could reverse or halt disease progression to the maximum extent, especially at the early stage of AKI. Recent studies showed that a wave of ferroptosis‐induced tubule cell death may trigger the early onset of AKI.^[^ ^4a^ ^]^ Synchronized ferroptosis secondarily recruits an inflammatory response that may trigger necroptosis or other forms of cell death in nearby tubules, indicating that ferroptosis is primarily responsible for early AKI damage. In this regard, the therapeutic outcomes may benefit from the prevention of the original wave of cell death caused by ferroptosis.

Currently, several small‐molecule ferroptosis inhibitors targeting iron metabolism and lipid metabolism were reported,^[^ ^24^ ^]^ which demonstrated robust nephroprotective effects against early kidney injury in various models of kidney disease.^[^ ^9a^ ^]^ However, the intrinsic limitations of small‐molecule drugs, such as low stability in the plasma, short half‐life (*t*_1/2_) time in circulation, and incomplete drug delivery routes, largely restrain their anti‐ferroptotic effect in the context of AKI. Engineered nanomaterials provide optimized delivery platforms that could improve the stability and solubility and prolong the circulation time of the encapsulated cargo. For those reasons, nanoplatforms that target the ferroptosis mechanism for ameliorating AKI have received considerable attention.^[^ ^16^, ^25^ ^]^ However, ferroptosis‐targeting nanoplatforms targeting singular ferroptosis inhibition mechanism may result in limited anti‐ferroptotic therapeutic effects. Because of the potential synergy between different mechanisms, it is crucial to design nanoplatforms with multifaceted ferroptosis inhibition mechanisms to enhance the therapeutic benefits in AKI.

In this work, we proposed an ultrasmall nanodot FerroD that could simultaneously remove overloaded iron ions and eliminate massive LPO to synergically enhance the anti‐ferroptotic ability. The in vitro experiments demonstrated that FerroD could rescue the CDDP‐treated HK‐2 cells by inhibiting ferroptosis. More importantly, mice treated with FerroD showed a robust anti‐ferroptotic ability to protect kidneys from the nephrotoxicity of CDDP in a long term. It is worth noting that the co‐assembly and disassembly of the FerroD confer two positive factors on the anti‐ferroptotic effect: i) TSD self‐assembly could prolong the DFO's half‐life while having little effect on its iron binding properties; ii) the physical encapsulation of Fer‐1 by TSD could improve its serum stability. The entrapped Fer‐1 could be released in ferroptotic renal cells by the coordination‐disassembly mechanism to scavenge the overproduced LPO. Beyond the context of AKI, we speculate that the FerroD is promising for the management of various ferroptosis‐related kidney diseases, such as ischemia‐reperfusion‐induced AKI,^[^ ^26^ ^]^ 5/6 nephrectomy‐induced chronic kidney disease (CKD),^[^ ^27^ ^]^ and lupus nephritis.^[^ ^28^ ^]^

In summary, we developed a nanomedicine‐based ferroptosis modulator, FerroD, equipped with a dual anti‐ferroptotic ability that provided long‐term nephroprotective effects in the kidneys of mice undergoing CDDP exposure. This study may shed new light on the design of nanomedicine targeting ferroptosis for the treatment of kidney diseases.

## 4 Experimental Section

### Synthesis of TSD

TPE‐Lys‐DFO (25 mg, 0.02 mmol, 1.0 eq.) and Boc‐Ser(tBu)‐N‐hydroxysuccinimide (NHS) (8 mg, 0.02 mmol, 1.0 eq.) were dissolved in anhydrous dimethyl sulfoxide (DMSO) (1 mL), followed by the additions of *N*,*N*‐diisopropylethylamine (10 µL, 0.06 mmol, 3.0 eq.). The reaction mixture was stirred at room temperature overnight. Deprotection cocktail 95:2.5:2.5 (v/v) trifluoroacetic acid (TFA):triisopropylsilane (TIS):H_2_O (1 mL) was added dropwise to a stirred solution for 3 h. The reaction mixture was concentrated under reduced pressure and then purified by reverse‐phase HPLC (Thermo Scientific C18 column) held at elution with a gradient of 20–95% CH_3_CN (0.1% CF_3_COOH) in water (0.1% CF_3_COOH) over 40 min, *t*_r_ = 18.59 min. This gave about 8 mg of the product as a white solid after lyophilization. ^1^H NMR (400 MHz, DMSO‐*d*_6_) *δ* 8.39 (s, 1H), 8.04 (s, 1H), 7.72 (d, *J* = 52.6 Hz, 2H), 7.23–6.61 (m, 19H), 4.38 (s, 2H), 3.68 (s, 1H), 3.49 (d, *J* = 7.9 Hz, 3H), 3.11–3.01 (m, 7H), 2.83–2.56 (m, 7H), 2.37–2.26 (m, 7H), 1.99 (s, 3H), 1.63–1.25 (m, 24H). High‐resolution mass spectrometry (HRMS)‐Electrospray ionisation (ESI): *m*/*z* calcd. for C_62_H_85_N_9_O_13_: 1163.6267, [M+H]^+^ found: 1164.5969; [1/2M+H]^+^ found: 582.7561.

### Western Blotting Assay

Kidney tissues from AKI mice were lysed using radio immunoprecipitation assay (RIPA) lysis buffer (50 mm Tris, 150 mm NaCl, 1% Triton X‐100, 1% sodium deoxycholate, and 0.1% sodium dodecyl sulfate (SDS)) and centrifuged at 10 000 rpm for 5 min at 4 °C. SDS‐polyacrylamide gel electrophoresis (SDS‐PAGE) was operated with 10 µg of proteins per well at a voltage of 100 V for 90 min. The proteins were transferred to a polyvinylidene difluoride membrane using wet transfer mode (Biorad) operated at 260 mA for 50 min. Membranes were incubated in primary antibodies overnight at 4 °C and then washed with tris buffered saline (TBS) containing 0.05% Tween‐20 five times each for 5 min. The PVDF membranes were then individually incubated with the following antibodies at the stated dilutions overnight at 4 °C: anti‐GPX4 and anti‐β‐actin The membranes were washed with TBST buffer (137 mm NaCl, 2.7 mm KCl, 16.5 mm Tris, pH 7.4, containing 0.1% Tween‐20) and incubated with secondary antibodies at 23 °C for 60 min. After another wash with tris buffered saline with tween (TBST), the detection of proteins was performed using enhanced chemiluminescence (ECL), and the results were analyzed and quantified by Image J.

### Estimation of Dissociation Constant (*K*_D_)

FeCl_3_ (25 µm) was premixed with ferrozine (75 µm; Sigma–Aldrich) in a 25 mm 2‐morpholinoethanesulphonic acid (MES)/Tris buffer at pH 7.0 containing 62.5 mm sodium ascorbate (Sigma–Aldrich). Then, precalculated amounts of DFO, TSD, and FerroD were mixed with Fe(III). The solutions were allowed to equilibrate for 24 h before measuring the absorbance at 562 nm. The concentration for 50% of maximal effect (EC_50_) values for each chelator were calculated by four‐parameter logistic regression (dotted curves) using GraphPad Prism v.8.0 after plotting the absorbance versus chelator concentrations, and the dissociation constant *K*_D_ for each complex was determined using the equation:

(1)

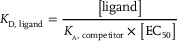

where *K*_A_ is an association constant, the ligand is the molecule originally bound to iron (i.e., ferrozine), and the competitor is the competing chelator (DFO, TSD, and FerroD).

### Pharmacokinetic Analysis

Mice were injected with FerroD (DFO 500 µg mL^−1^) and blood was collected via orbital at the following time points: 0, 1, 3, 5, 10, 30, 60, 120, 180, and 240 min. Blood was stored in an ice box to prevent clotting. TSD was quantified using HPLC. Pharmacokinetic analysis of TSD was performed using the two‐compartment model to estimate elimination (*t*_1/2_) blood half‐life values.

### DFT Calculation

The geometry of all complexes was optimized through XTB software.^[^ ^29^ ^]^ The single point energies were calculated with the Gaussian 16 software at B3LYP/Def2‐SVP theoretical level. The DFT‐D3 dispersion correction with BJ‐damping.^[^ ^30^ ^]^ was applied to correct the weak interaction to improve the calculation accuracy. Meanwhile, the basis set superposition error (BSSE) was dealt with boys and by the Bernardi's counterpoise method.^[^ ^31^ ^]^

### Blood Analysis

Blood was collected from the orbital in living C57BL/6 mice under isoflurane anesthesia at *t* = 4, 8, 12 day post treatment with CDDP (15 mg kg^−1^ body weight) and saline‐treated mice. The collected blood samples were centrifuged for 20 min at 3500 rpm. The sCr, BUN, Kim‐1, and NGAL were determined using commercial kits according to the manufacturer's protocol.

### The MRI of CDDP‐Induced AKI Mouse Model

The in vivo MRI of CDDP‐induced AKI mice was conducted by a 9.4 T scanner (Bruker). A prefixed catheter was used in the mouse tail vein to acquire pre‐ and postcontrast *T*_1_. After sequential scanning of pre‐contrast *T*_1_‐weighted MRI using the same in‐plane geometries, the Art–Gd probe (1.12 × 10^3^ µm of Gd) was injected intravenously from outside the scanner, while the mouse was kept anesthetized and left steady. Then, the postcontrast *T*_1_‐weighted MRI was acquired at different postinjection times through the same sequence parameters. The imaging sequence for *T*_1_‐weighted MRI was RARE‐VTR pulse using the following parameters: echo time = 8.5 ms, effective TE = 8.5 ms, rare factor = 4, number of experiments = 6, multiple repetition time = 5500, 3000, 1500, 800, 400, 327.103 ms; number of averages = 1, number of repetitions = 1, matrix = 256 × 256. Scan time = 9 min 13 s 300 ms.

### Evaluating the Therapeutic Efficacy in CDDP‐Induced AKI Mice

200 µL of saline, Fer‐1 (0.5 mg mL^−1^) + DFO (2.5 mg mL^−1^), Fer‐1@TD (1.5 mg mL^−1^), and FerroD (1.5 mg mL^−1^) were i.p. injected 1 h before CDDP injection and 24 and 48 h after CDDP injection. Subsequently, *T*_1_‐weighted MRI was conducted to acquire pre‐ and postcontrast *T*_1_ before and after i.v. injection of the Art–Gd probe (1.12 × 10^3^ µm of Gd) at *t* = 4, 8, 12 day post‐treatment with CDDP (15 mg kg^−1^). The blood samples of AKI mice at different time points post‐treatment with CDDP were collected for analysis of kidney function. Kidney tissues from each group were collected at different time points post‐treatment with CDDP for histological examination.

### Statistical Analysis

All the quantitative data were expressed as mean ± standard deviation (s.d.). Comparisons were analyzed using a two‐tailed unpaired Student's *t*‐test or a one‐way analysis of variance (ANOVA). GraphPad Prism (v.8.0), FlowJo (v10.6.2), and ImageJ (v1.8.0) were used for statistical analysis and figure production. The statistical significance was indicated as ns (no significance), ^*^ *P* < 0.05, ^**^ *P* < 0.01, and ^***^ *P* < 0.001.

## Conflict of Interest

The authors declare no conflict of interest.

## Author Contributions

F. Z. and Y. Q. contributed equally to this work. Z.Z. and F.Z. conceived and designed the project. F.Z., Y.Q., J.Y., Y.L., and S.N. synthesized and characterized the organic molecules. F.Z., H.S., H.X., J.C., and S.C. performed the MRI and the data processing. C.Y. performed the density functional theory calculations. F.Z. and Z.Z. analyzed and discussed the results. F.Z., L.T., and Z.Z. analyzed the data and co‐wrote the paper. Z.Z. supervised the entire project. All the authors have approved the final version.

## Supporting information

Supporting Information

## Data Availability

The data that support the findings of this study are available in the Supporting Information of this article.
