# Supplementary material for: Ultrasmall Nanodots with Dual Anti‐Ferropototic Effect for Acute Kidney Injury Therapy
Source: Adv Sci (Weinh). 2024 Aug 19;11(39):2403305. doi: 10.1002/advs.202403305 (PMC11497046; doi:10.1002/advs.202403305)
Supplement: Supplementary file 1 — Supporting Information [file ADVS-11-2403305-s001.docx]

**Ultrasmall nanodots with dual-antiferropototic effect for acute kidney injury therapy**

Fantian Zeng^1,6^, Yatong Qin^1,6^, Sureya Nijiati^1^, Yangtengyu Liu^2^, Jinmin Ye^1^, Huaxiang Shen^1^, Jiayuan Cai^1^, Hehe Xiong^1^, Changrong Shi^1,3^, Longguang Tang^4*^, Chunyang Yu^5^*, Zijian Zhou^1^*

^1^State Key Laboratory of Vaccines for Infectious Diseases, Xiang An Biomedicine Laboratory, School of Public Health, Shenzhen Research Institute of Xiamen University, Xiamen University, Xiamen 361102, China.

^2^Department of Rheumatology and Immunology, Xiangya Hospital, Central South University, Changsha 410008, China.

^3^Departments of Diagnostic Radiology, Surgery, Chemical and Biomolecular Engineering, and Biomedical Engineering, Yong Loo Lin School of Medicine and College of Design and Engineering, National University of Singapore, Singapore, 119074, Singapore.

^4^Gaozhou People's Hospital, Maoming, 525200 China.

^5^School of Chemistry and Chemical Engineering, State Key Laboratory of Metal Matrix Composites, Shanghai Jiao Tong University, 800 Dongchuan Road, Shanghai, 200240, China.

^6^F. Z. and Y. Q. contributed equally to this work.

***Correspondence to:**

Zijian Zhou (Ph.D.), E-mail: [zhouz@xmu.edu.cn](mailto:zhouz@xmu.edu.cn)

Chunyang Yu (Ph.D.), E-mail: chunyangyu@sjtu.edu.cn

Longguang Tang (Ph.D.), E-mail: tanglongguang@zju.edu.cn

**Materials and Methods**

**Materials**

Deferoxamine mesylate (DFO) and 2-(4-(1,2,2-triphenylvinyl)phenoxy)acetic acid were purchased from Bide Pharmatech Ltd. Boc-O-tert-butyl-L-serine, N-alpha-(tert-Butoxycarbonyl)-L-lysine and N-hydroxysuccinimide (NHS) were purchased from Aladdin. 4-(4,6-Dimethoxy-1,3,5-triazin-2-yl)-4-methylmorpholin-4-ium chloride (DMTMM), N, N-Diisopropylethylamine (DIPEA), and cisplatin were purchased from Energy Chemical. Anti-glutathione peroxidase (GPX4, ab125066) was purchased from Abacm. Anti-kidney injury molecule-1 (Kim-1, GTX85068) was purchased from GeneTex. β-Actin (8H10D10) was purchased from Cell Signaling Technology. FerroOrange was purchased from Maokang Biotechnology The creatinine, blood urea nitrogen and neutrophil gelatinase-associated lipocalin (NGAL) assay kits were purchased from Ponstar Biotech Co.,Ltd.

**Cell culture and animal model**

Human kidney 2 (HK-2) cells were acquired from the American Type Culture Collection and cultured in Dulbecco’s modified Eagle’s medium (DMEM) containing 10% heat-inactivated fetal bovine serum (FBS) and supplemented to a final concentration with L-glutamine (2 mM), penicillin (50 U/ml), streptomycin (50 μg/ml). All animal experiments were carried out in accordance with the Guide Protocol of Laboratory Animals, approved by the Ethics Committee of the Xiamen University. Four- to six-week-old female C57BL/6 mice were ordered from the Xiamen University Animal Center. To establish the AKI mouse model, C57BL/6 mice were treated with cisplatin (15 mg kg^-1^ body weight, i.p. injection).

**Synthesis of** **TPE-Lys-boc**

A round-bottom flask was charged with 2-(4-(1,2,2-triphenylvinyl)phenoxy)acetic acid (TPE-COOH) (250 mg, 0.61 mmol, 1.0 eq), dicyclohexylcarbodiimide (DCC) (106 mg, 0.92 mmol, 1.5 eq), and NHS (190 mg, 0.92 mmol, 1.5 eq), and anhydrous dichloromethane (5 mL) was stirred at 0 °C for 1 h. After overnight stirring at room temperature. The reaction mixture was filtered through a bed of celite. The celite was washed with additional dichloromethane (3 × 20 mL). The filtrate was concentrated to give TPE-NHS ester as a white solid. A round-bottom flask charged with TPE-NHS ester (100 mg, 0.20 mmol, 1.0 eq), N-alpha-(tert-Butoxycarbonyl)-L-lysine (48 mg, 0.20 mmol, 1.0 eq), DIPEA (77 µL, 0.60 mmol, 3.0 eq), and anhydrous DMSO (4 mL). The reaction mixture was stirred at room temperature overnight and monitored with TLC. The resulting mixture was extracted with ethyl acetate. The organic phase was dried over anhydrous Na_2_SO_4_ and evaporated to give a yellow liquid. The crude product was purified by flash chromatography using petroleum ether:ethyl acetate (3:1, v/v) as fluent to afford product as yellow-white powder. ^1^H NMR (400 MHz, DMSO-*d*_6_) *δ* 8.08 – 7.77 (m, 1H), 7.14 – 6.65 (m, 20H), 4.51 (q, *J* = 6.3, 5.1 Hz, 1H), 3.78 (s, 2H), 3.12 (d, *J* = 5.9 Hz, 2H), 1.71 – 1.07 (m, 15H). HRMS (ESI): m/z calcd for C_39_H_42_N_2_O_6_: 634.3043, [M + K]^+^ found: 673.2404; [M + Na]^+^ found: 657.2718; [2M + Na]^+^ found: 1291.6014.

**Synthesis of TPE-lys-DFO (TD)**

TPE-Lys-boc (50 mg, 0.08 mmol, 1.0 eq), DFO (51 mg, 0.08 mmol, 1.0 eq), and DMTMM (70 mg, 0.20 mmol, 3.0 eq) were dissolved in anhydrous DMSO (2 mL). The reaction mixture was stirred at room temperature overnight and purified by reverse phase HPLC (Thermo Scientific C18 column) held at elution with a gradient of 20%-95% CH_3_CN (0.1% CF_3_COOH) in water (0.1% CF_3_COOH) over 40 min, tr = 19.32 min. This gave about 10 mg of the product as a white solid after lyophilization. HRMS (ESI): m/z calcd for C_59_H_80_N_8_O_11_: 1076.5947, [M + H]^+^ found: 1077.5356.

**Synthesis of** **Boc-Ser(tBu)-NHS**

NHS (0.73 g, 6.3 mmol, 1.05 eq) was added to a solution of (2S)-3-(tert-butoxy)-2-(tert-butoxycarbonylamino)-propanoic acid (1.57 g, 6.0 mmol, 1.0 eq) in ethyl acetate:1,4-dioxane (1:1, v/v) (18 mL) at 0 °C. DCC (1.30 g, 6.3 mmol, 1.05 eq.) was added in one portion. Then the reaction mixture was stirred at room temperature for 3 h. The reaction mixture was filtered through a Celite pad and concentrated. The crude product was redissolved in ethyl acetate (20 mL) and washed sequentially with H_2_O (20 mL) and saturated NaCl (20 mL). The organic layer was dried over anhydrous Na_2_SO_4_ and concentrated to afford crude Boc-Ser(tBu)-NHS as a white powder of sufficient purity. ^1^H NMR (400 MHz, Chloroform-*d*) δ 5.43 (dd, *J* = 38.6, 10.6 Hz, 1H), 3.94 – 3.53 (m, 2H), 2.82 (d, *J* = 39.6 Hz, 4H), 1.50 (s, 9H), 1.24 (s, 9H).


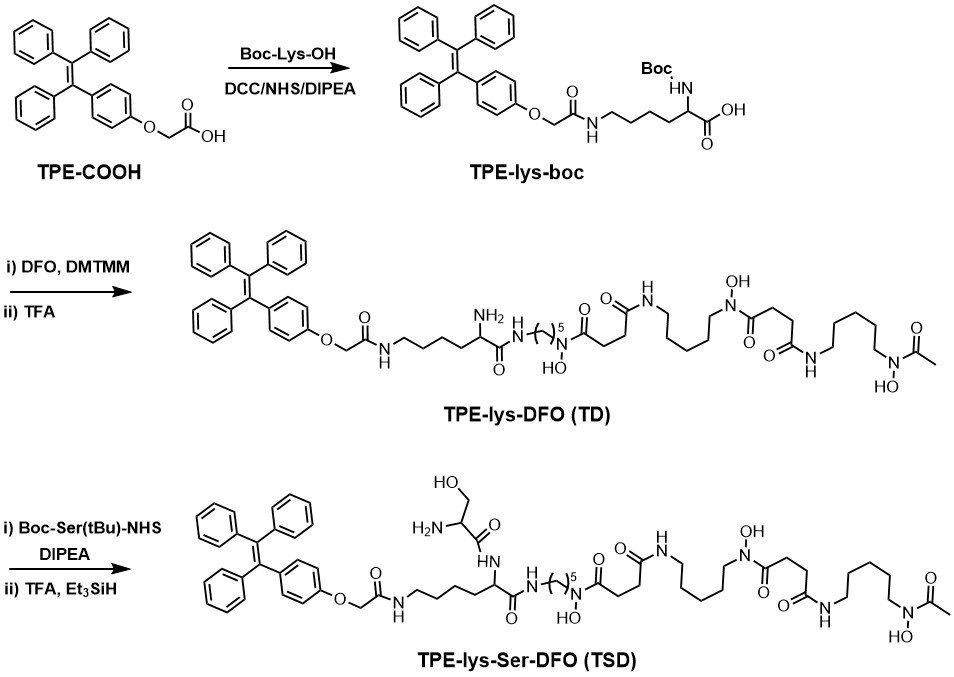


Figure S1 | The synthetic route of TSD. DCC: Dicyclohexylcarbodiimide, NHS: N-hydroxysuccinimide ester, DIPEA: Diisopropyl triethylamine, DMTMM: 4-(4,6-Dimethoxy-1,3,5-triazin-2-yl)-4-methylmorpholinium chloride, TFA: Trifluoroacetic acid.


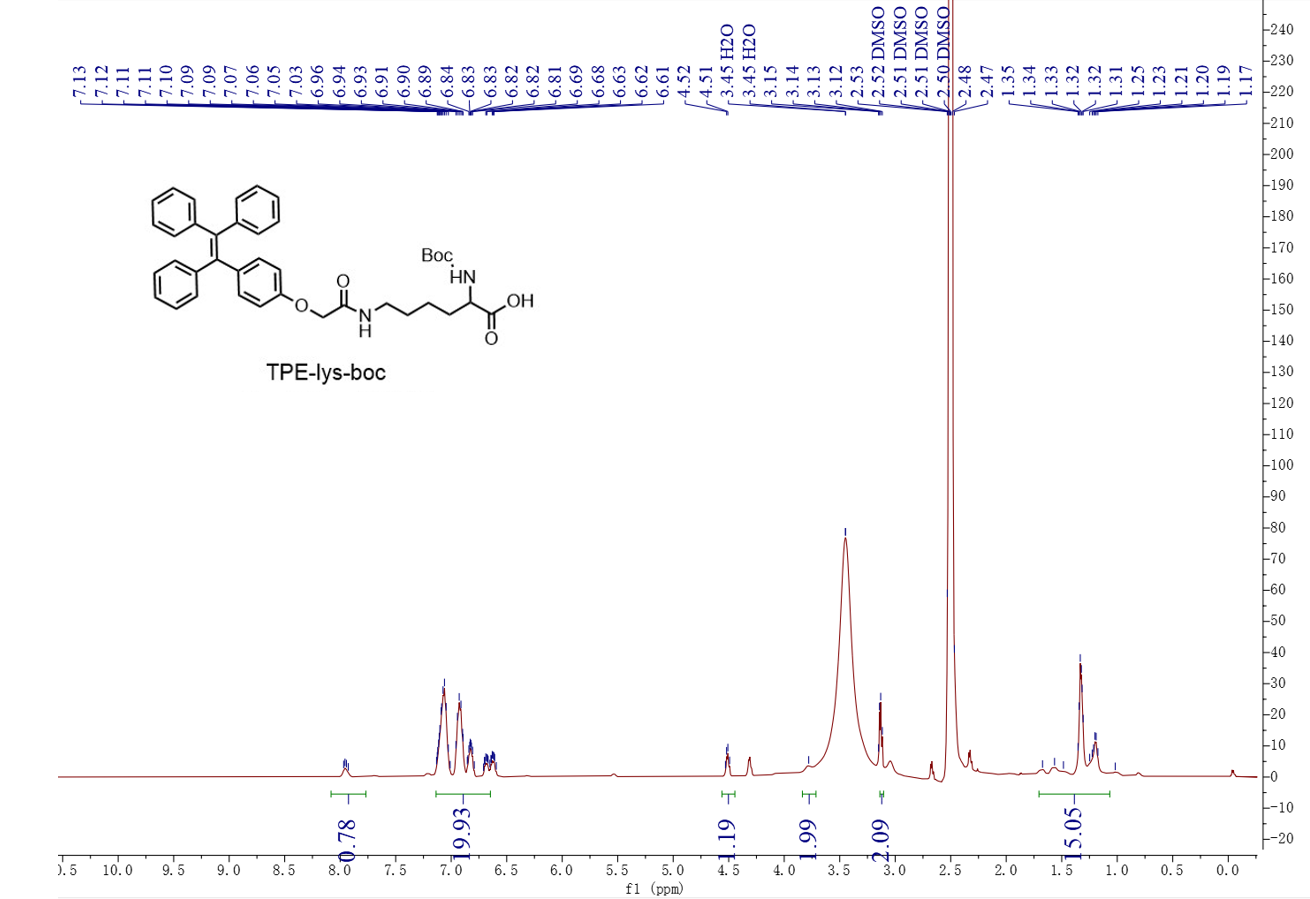


**Figure S2 | ^1^H NMR spectrum of TPE-lys-boc.** ^1^H NMR (400 MHz, DMSO-*d*_6_) δ 8.08 – 7.77 (m, 1H), 7.14 – 6.65 (m, 20H), 4.51 (q, *J* = 6.3, 5.1 Hz, 1H), 3.78 (s, 2H), 3.12 (d, *J* = 5.9 Hz, 2H), 1.71 – 1.07 (m, 15H).


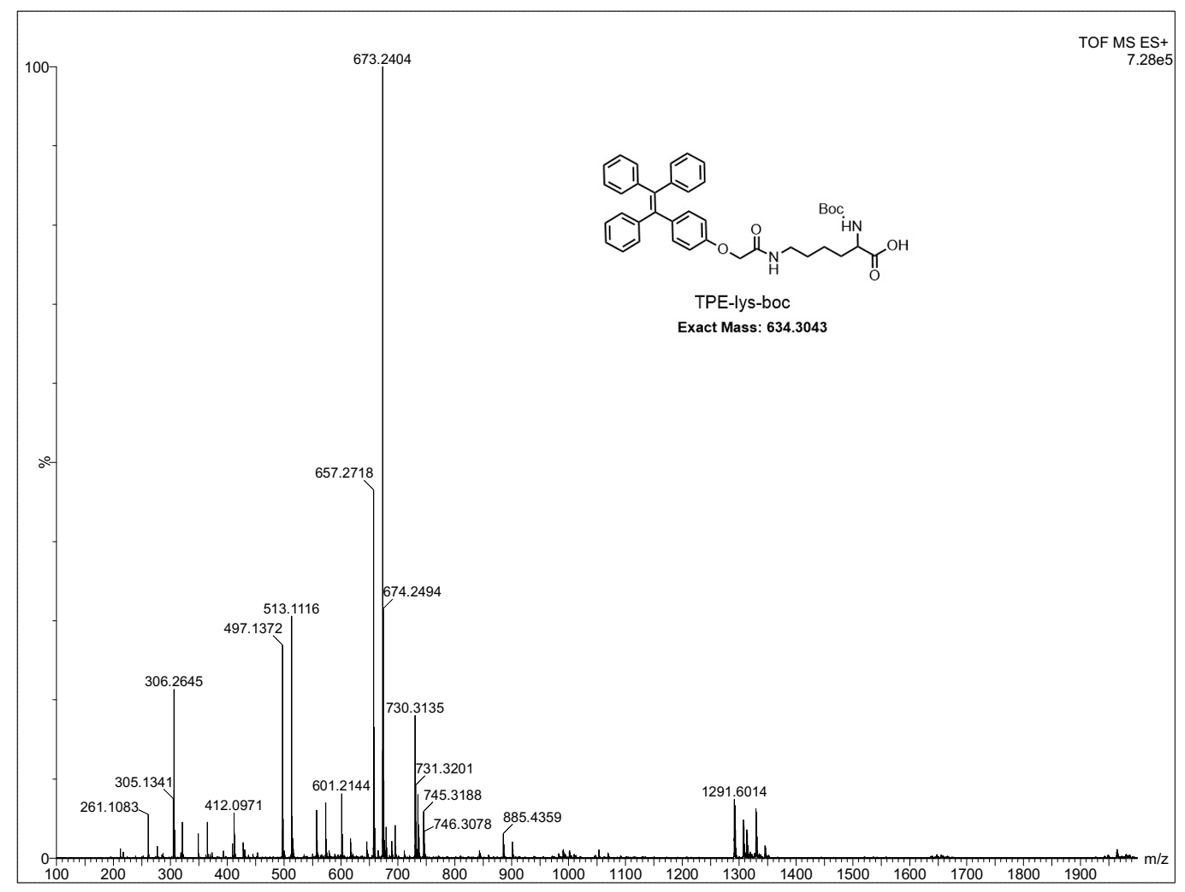


**Figure S3 | MS spectrum of TPE-lys-boc.** HRMS (ESI): m/z calcd for C_39_H_42_N_2_O_6_: 634.3043, [M+K]^+^ found: 673.2404; [M+Na]^+^ found: 657.2718; [2M+Na]^+^ found: 1291.6014.


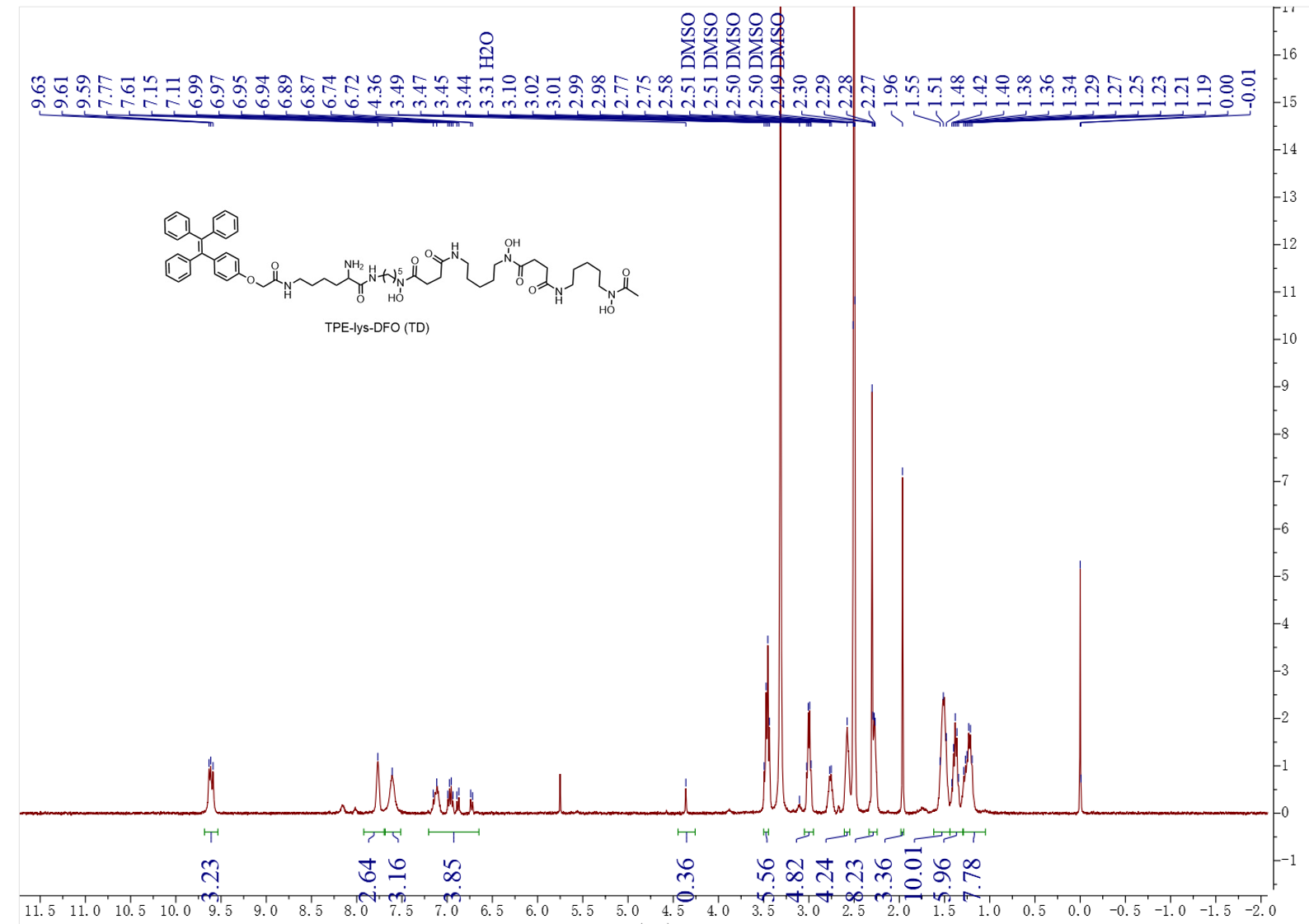


**Figure S4 | ^1^H NMR spectrum of TD.** ^1^H NMR (400 MHz, DMSO-*d*_6_) δ 9.68 – 9.54 (m, 3H), 7.77 (s, 3H), 7.61 (s, 3H), 7.20 – 6.65 (m, 4H), 3.46 (d, *J* = 7.6 Hz, 6H), 3.00 (q, *J* = 6.6 Hz, 5H), 2.58 (s, 4H), 2.29 (d, *J* = 9.3 Hz, 8H), 1.96 (s, 3H), 1.50 (d, *J* = 12.9 Hz, 10H), 1.37 (q, *J* = 7.3 Hz, 6H), 1.24 (dq, *J* = 15.2, 7.5 Hz, 8H).


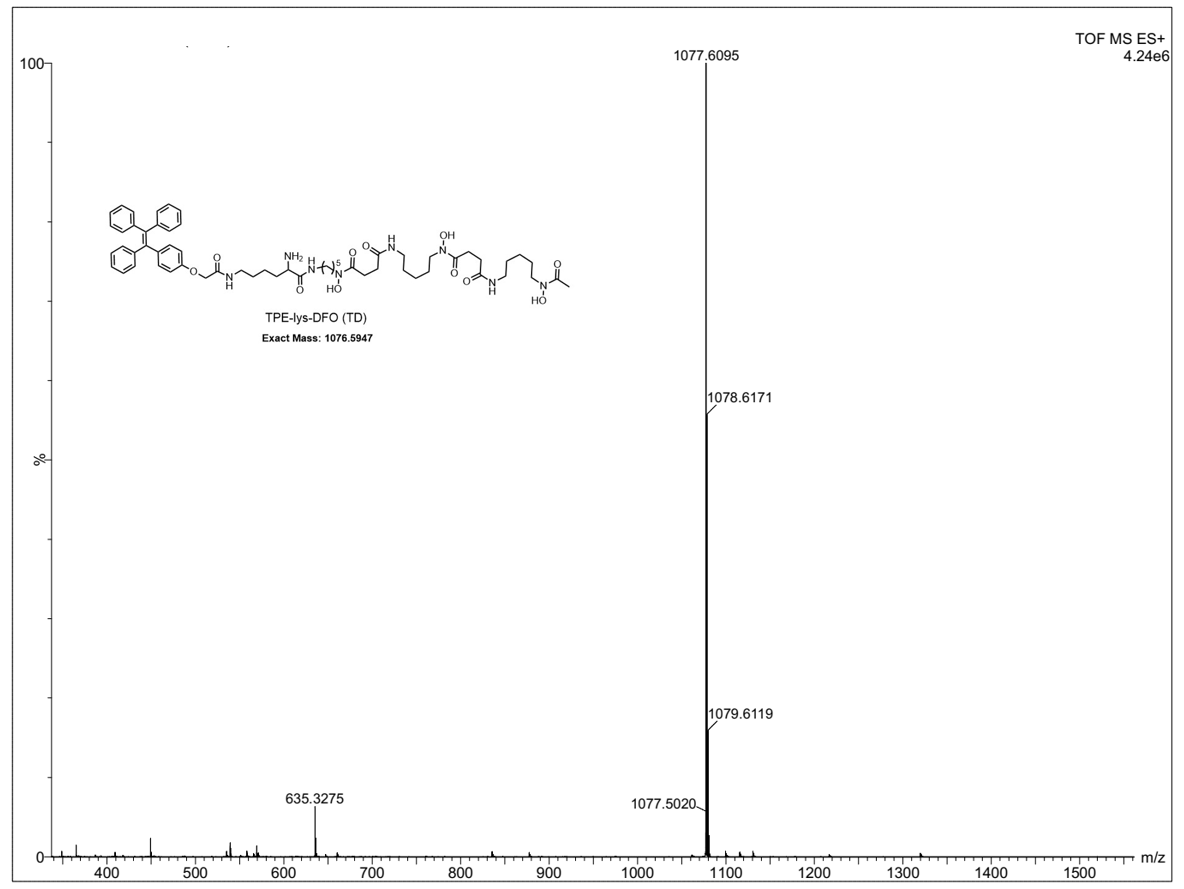


**Figure S5 | MS spectrum of TD.** HRMS (ESI): m/z calcd for C_59_H_80_N_8_O_11_: 1076.5947, [M+H]^+^ found: 1077.5356.


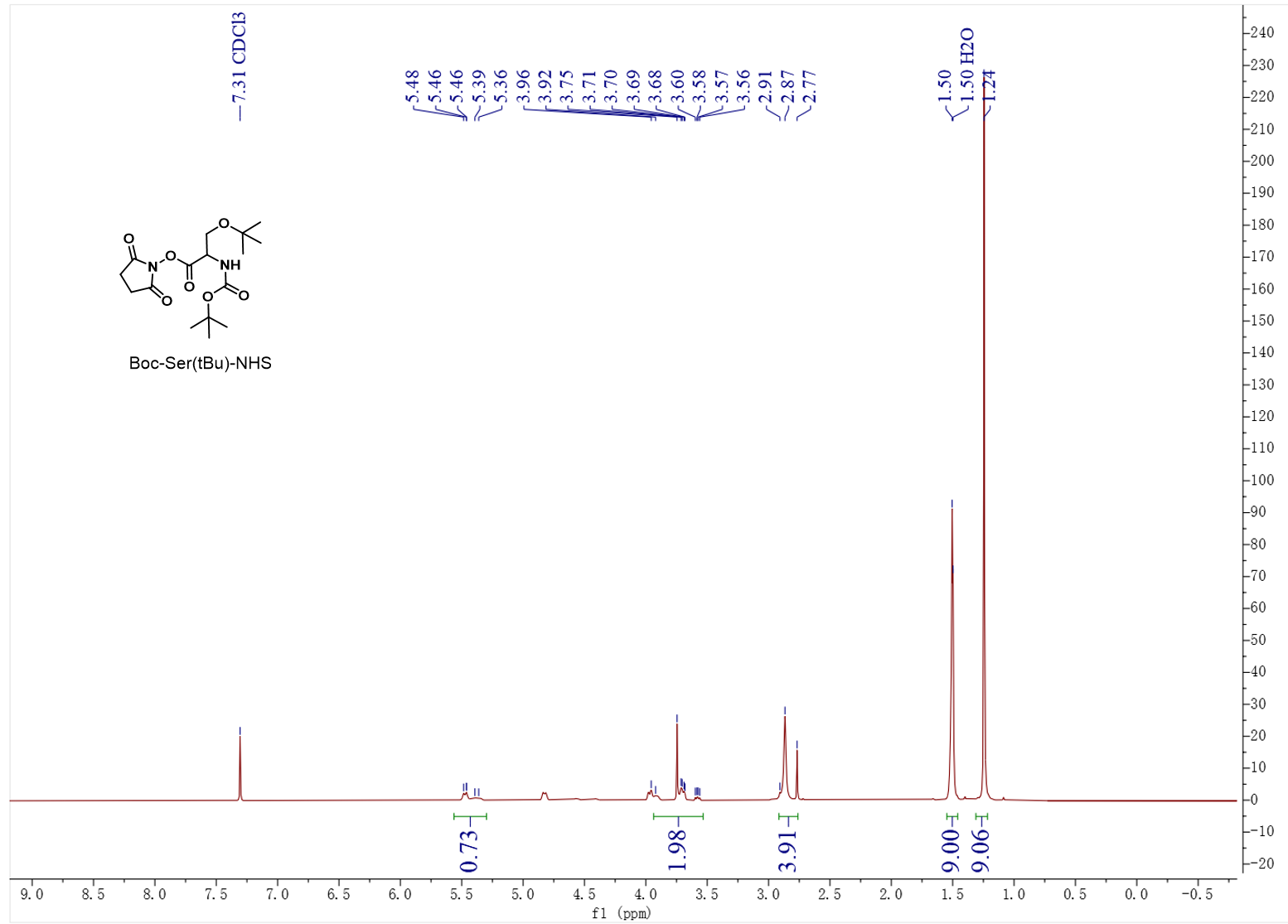


**Figure S6 | ^1^H NMR spectrum of Boc-Ser(tBu)-NHS.** ^1^H NMR (400 MHz, Chloroform-*d*) δ 5.43 (dd, *J* = 38.6, 10.6 Hz, 1H), 3.94 – 3.53 (m, 2H), 2.82 (d, *J* = 39.6 Hz, 4H), 1.50 (s, 9H), 1.24 (s, 9H).

**Figure S7 | MS spectrum of Boc-Ser(tBu)-NHS.** HRMS (ESI): m/z calcd for C_16_H_26_N_2_O_7_: 358.39, [M+Na]^+^ found: 381.16; [2M+Na]^+^ found: 739.34


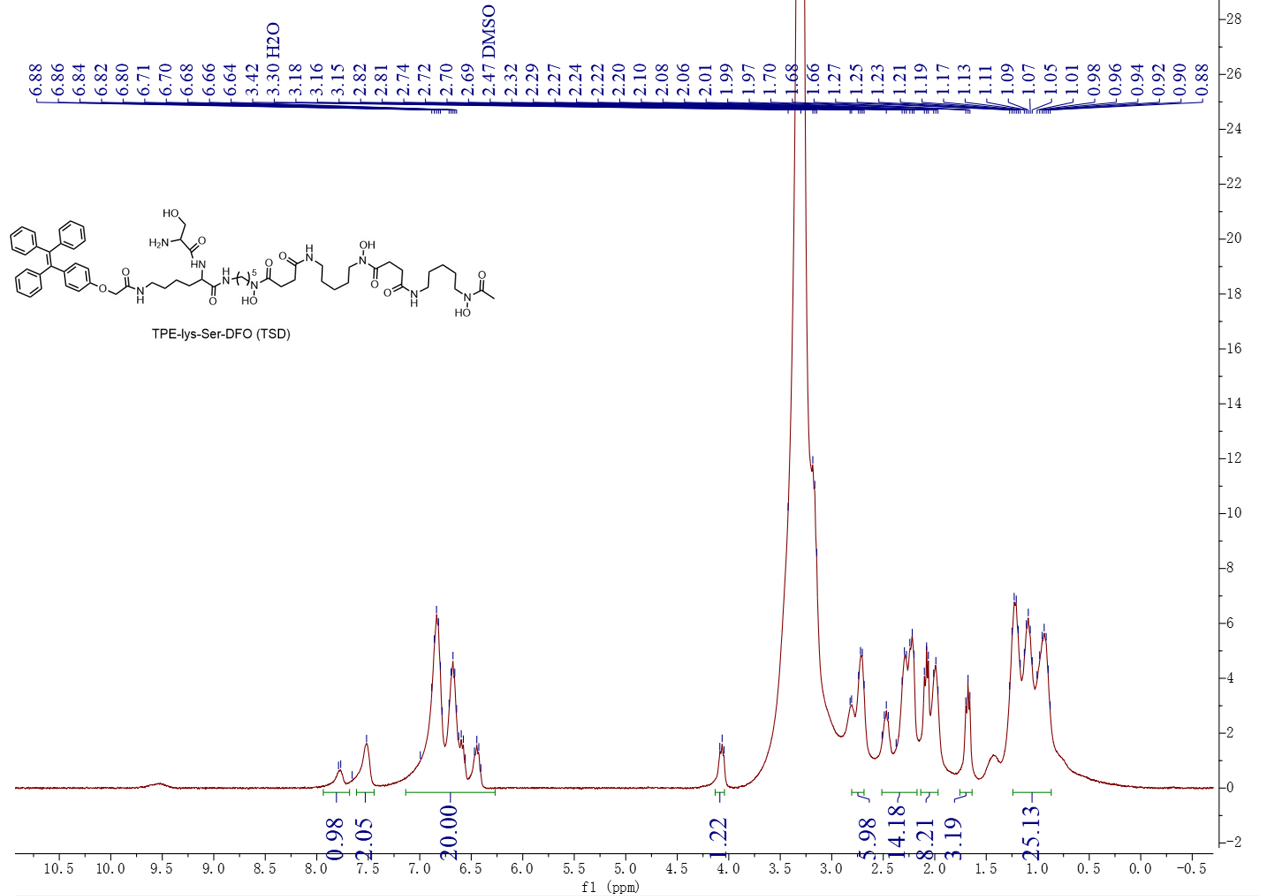


**Figure S8 | ^1^H NMR spectrum of TSD.** ^1^H NMR (400 MHz, DMSO-*d*_6_) δ 7.94 – 7.68 (m, 1H), 7.52 (s, 2H), 7.14 – 6.27 (m, 20H), 4.13 – 4.04 (m, 1H), 2.81 – 2.69 (m, 6H), 2.51 – 2.17 (m, 14H), 2.14 – 1.97 (m, 8H), 1.76 – 1.64 (m, 3H), 1.24 – 0.87 (m, 25H).


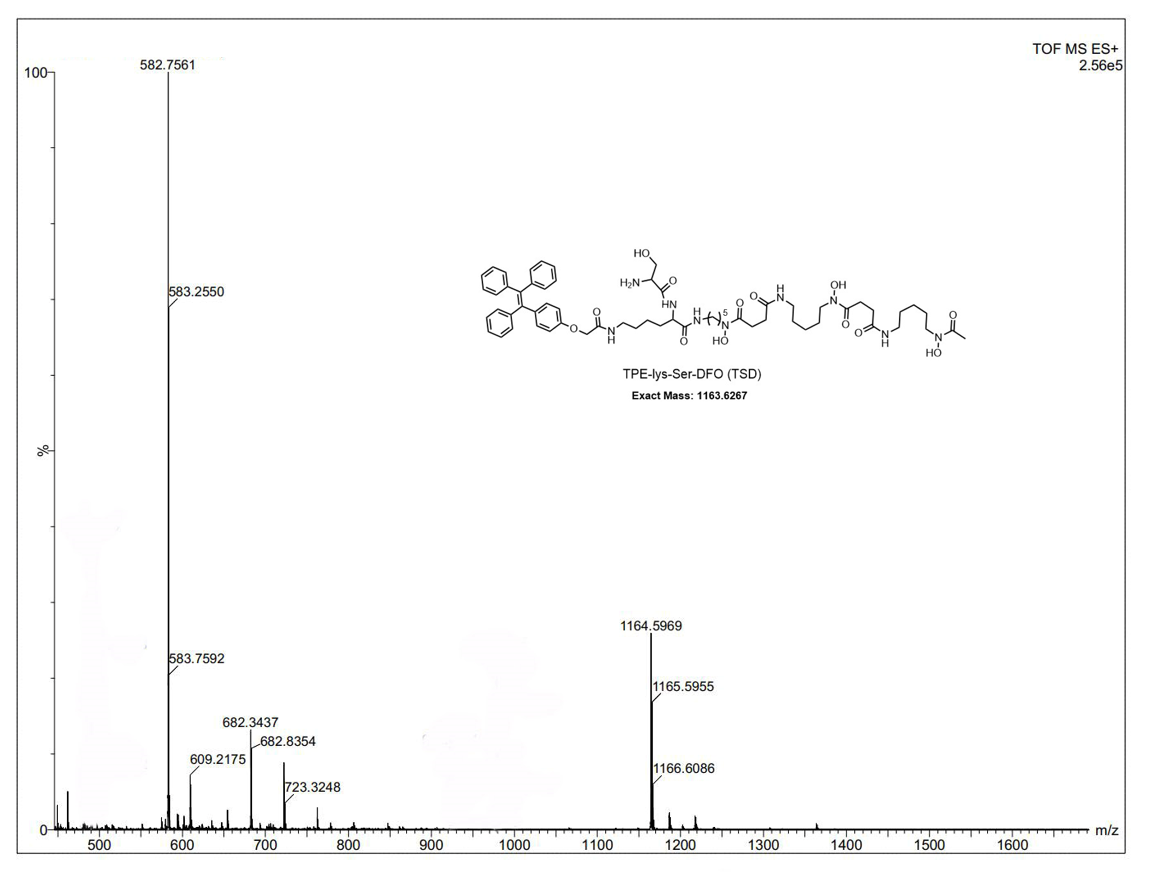


**Figure S9 | MS spectrum of TSD.** HRMS (ESI): m/z calcd for C_62_H_85_N_9_O_13_: 1163.6267, [M+H]^+^ found: 1164.5969; [1/2M+H]^+^ found: 582.7561.


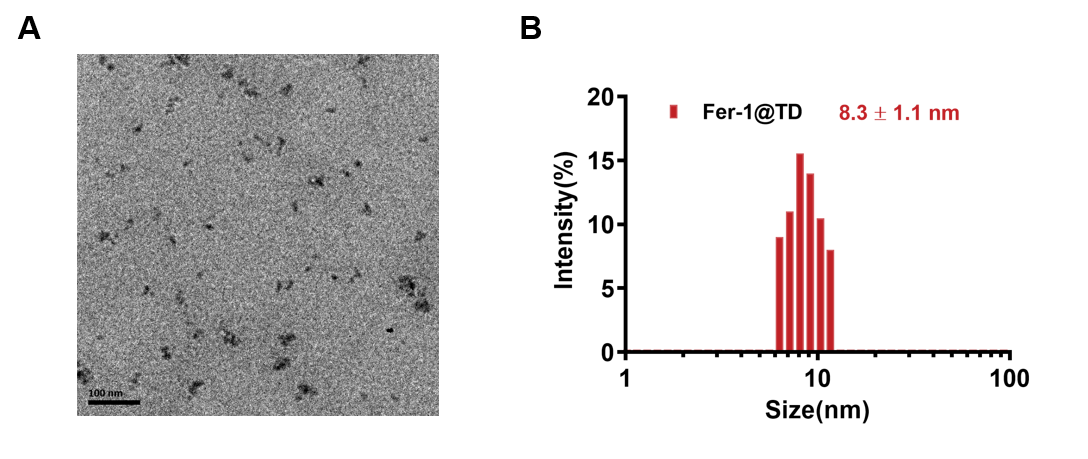


Figure S10 | (A) TEM images and (B) the DLS measurements of Fer-1@TD.


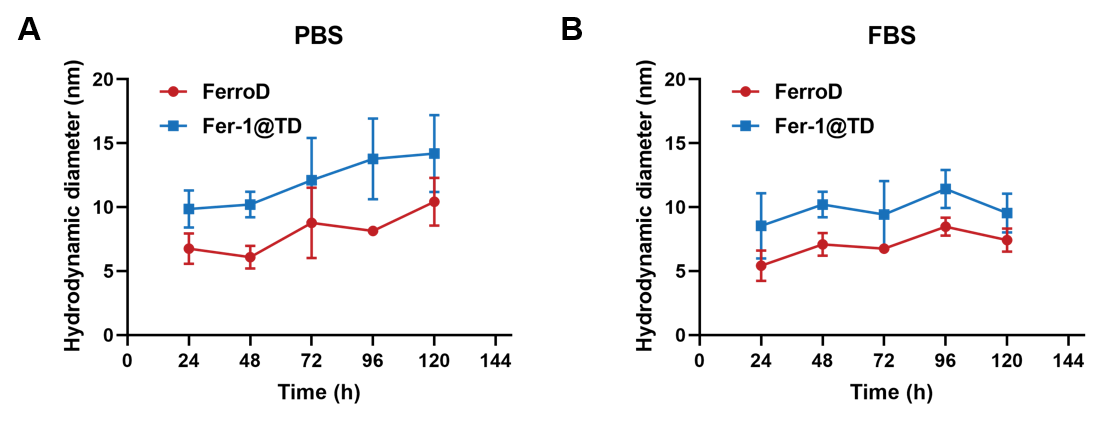


**Figure S11 |** (A, B) The stability of FerroD and Fer-1@TD in PBS and fetal bovine serum (FBS).


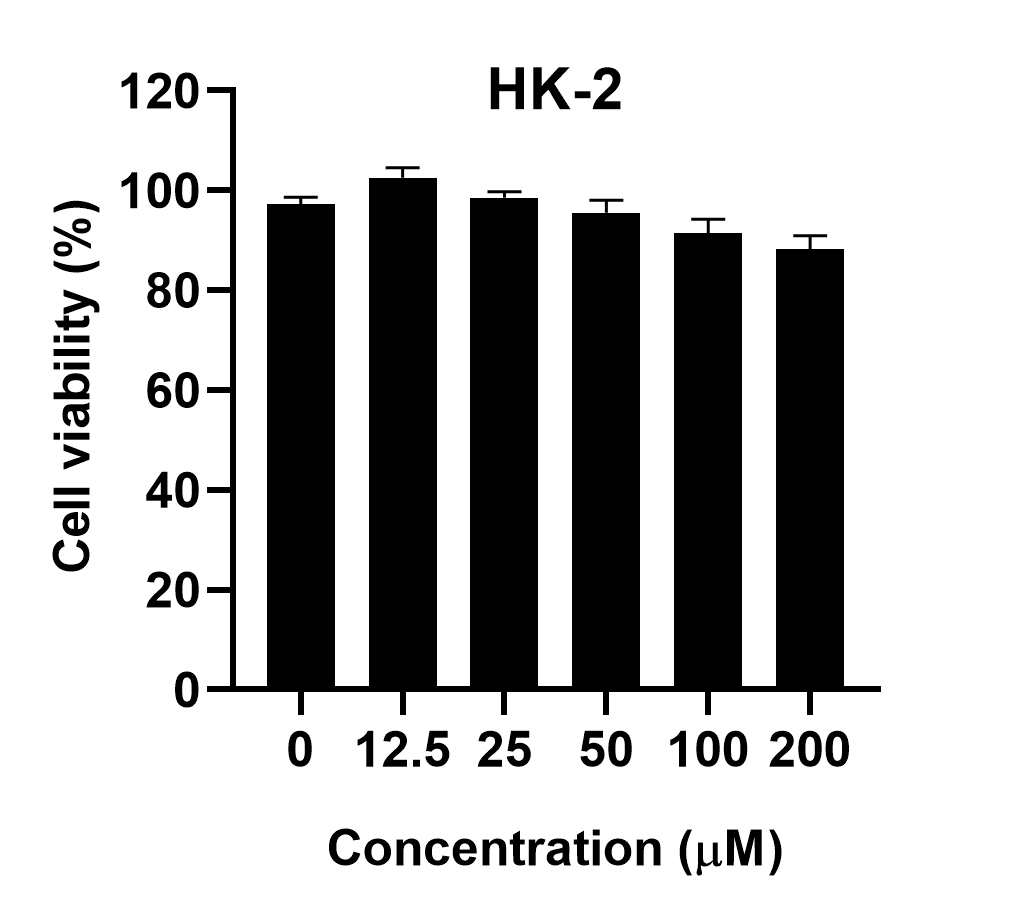


Figure S12 | The cell viability profiles of HK-2 cells after incubation with different concentrations of FerroD (for TSD).


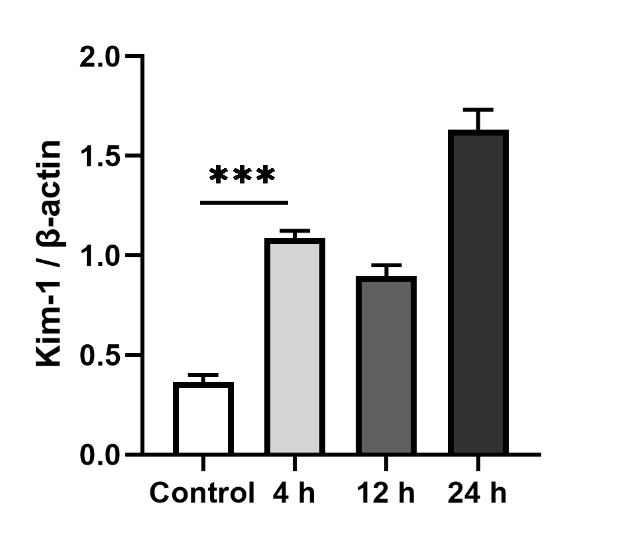


Figure S13 |The semi-quantitative analysis of Kim-1 levels in the HK-2 cells treated with CDDP at different time points. ****P* < 0.001.


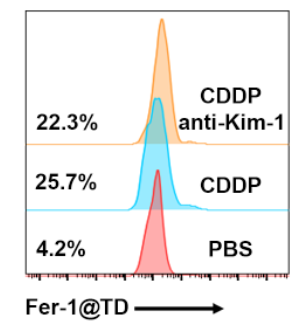


Figure S14 | The cellular uptake of Fer-1@TD in PBS-treated HK-2 cells, CDDP-induced HK-2 cells, and CDDP-induced HK-2 cells before treatment with anti-Kim-1 antibody.

.


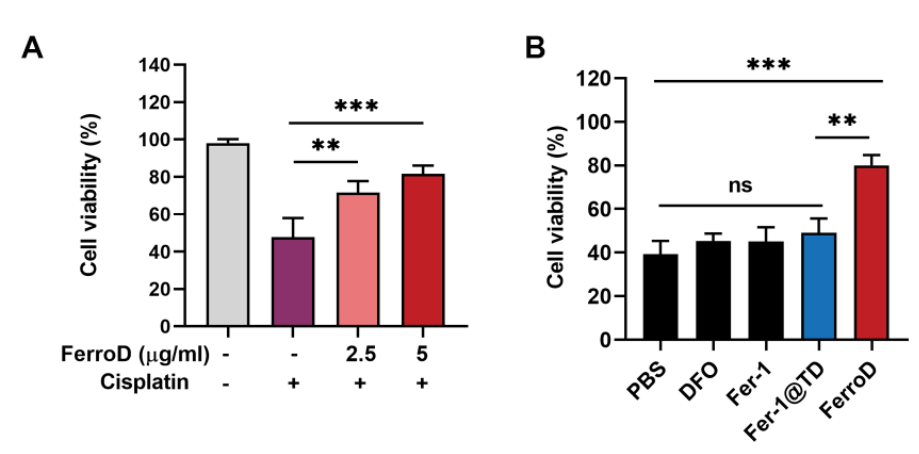


Figure S15 | (A) The cell viability of CDDP-induced HK-2 cells after incubating with FerroD at various concentrations. (B) The cell viability of CDDP-induced HK-2 cells after incubating with different pretreatment.


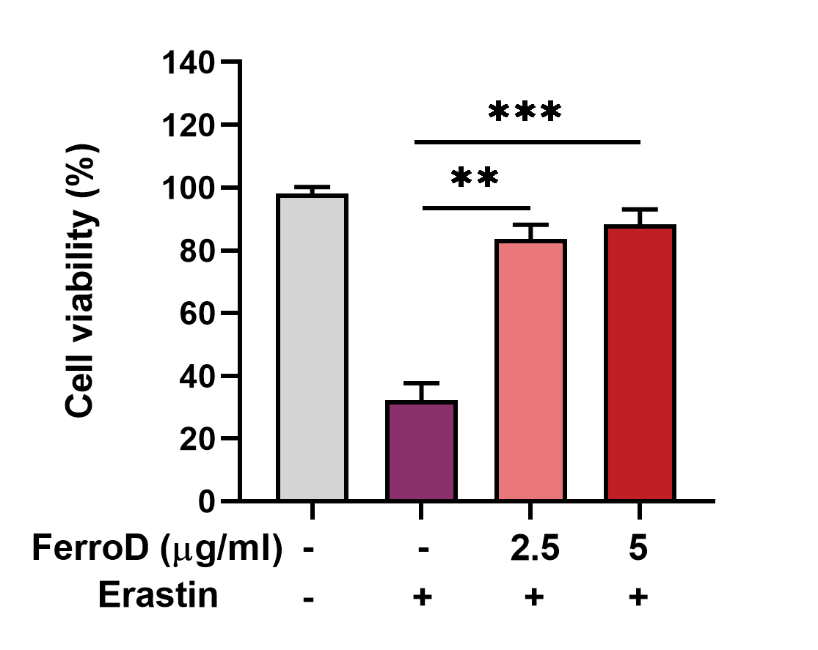


Figure S16 | The cell viability of Erastin-treated HK-2 cells after incubating with FerroD at various concentrations. ***P* < 0.01 and *** *P* < 0.001.


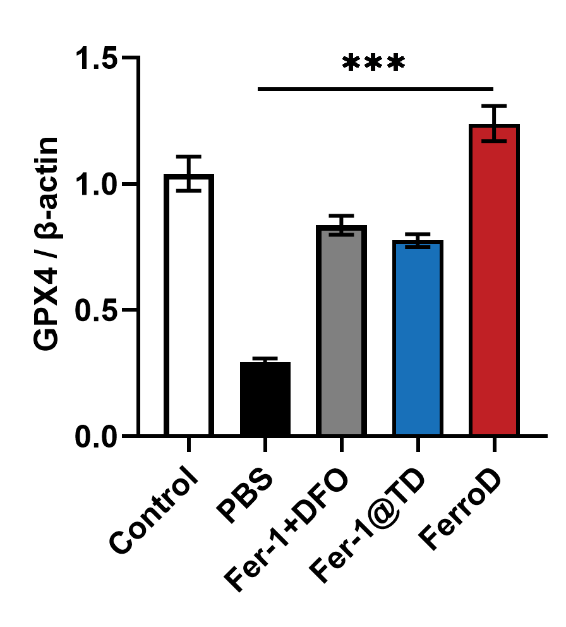


Figure S17 |The semi-quantitative analysis of GPX4 levels in the CDDP-induced HK-2 cells after different treatments. ****P* < 0.001.


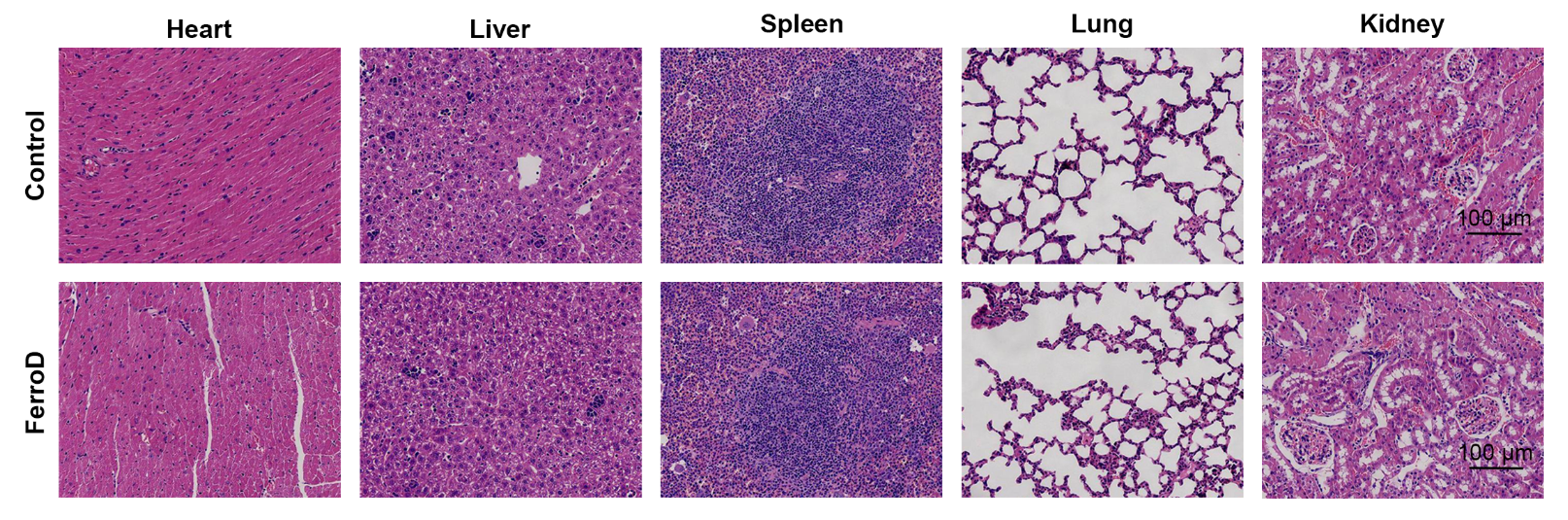
 Figure S18 | The H&E staining of major organs after the FerroD treatments. The major organs were dissected at 24 h after i.v. injection of the FerroD in normal mice. Scale bar = 100 µm for all images


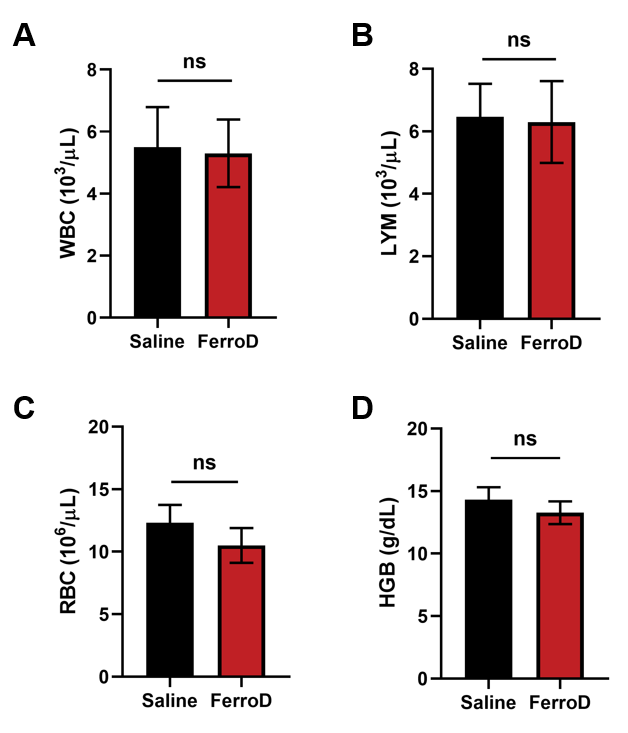


Figure S19 | Blood routine analysis of saline- and FerroD-treated mice (n=3 per group). Data are means ± SD, ns: not significant.


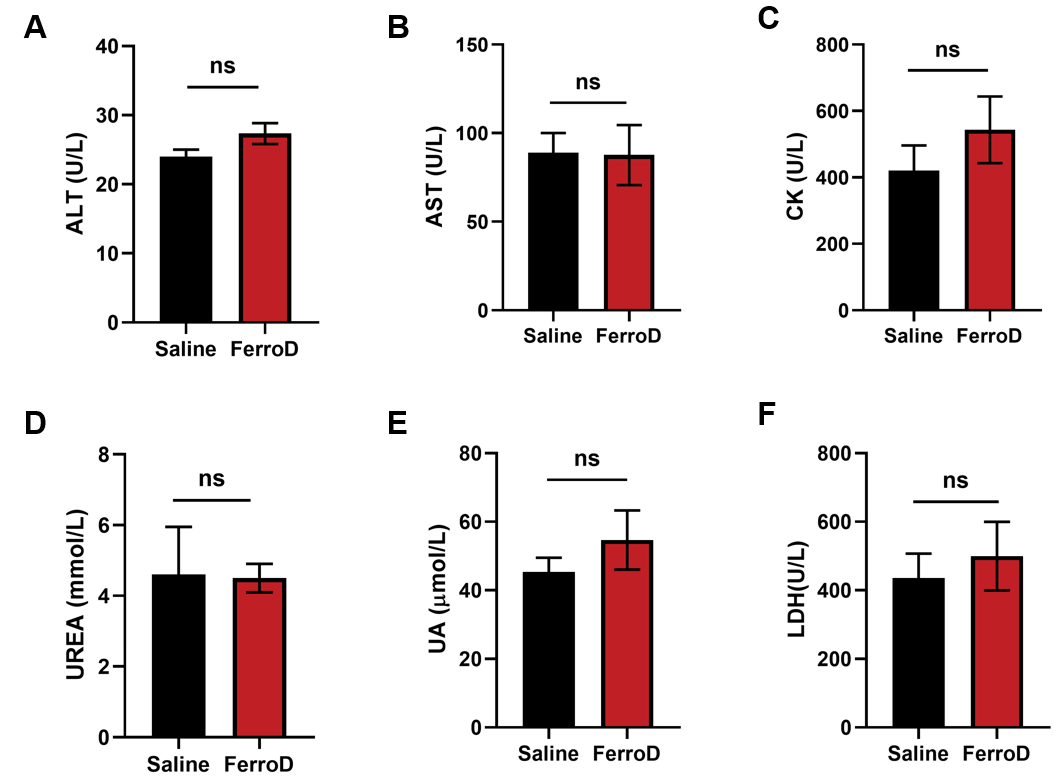


Figure S20 | Blood biochemical analysis of saline- and FerroD-treated mice (n=3 per group). Data are means ± SD, ns: not significant.


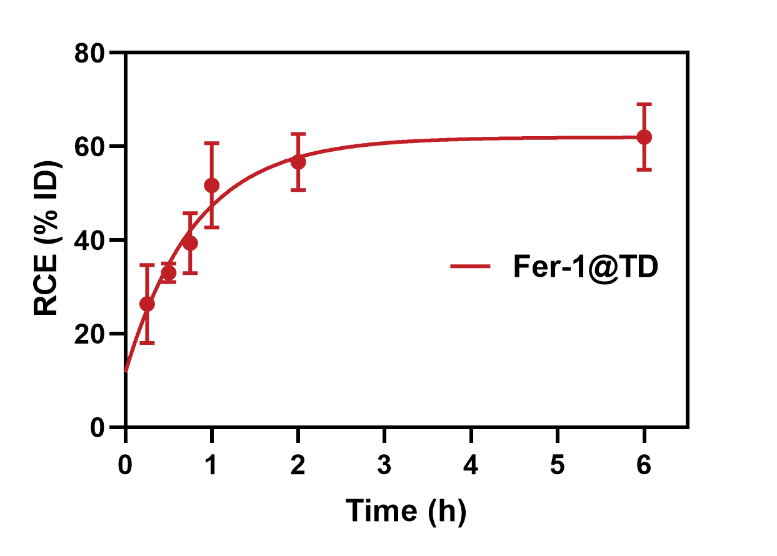


Figure S21 | The RCE of Fer-1@TD at different post-injection time.


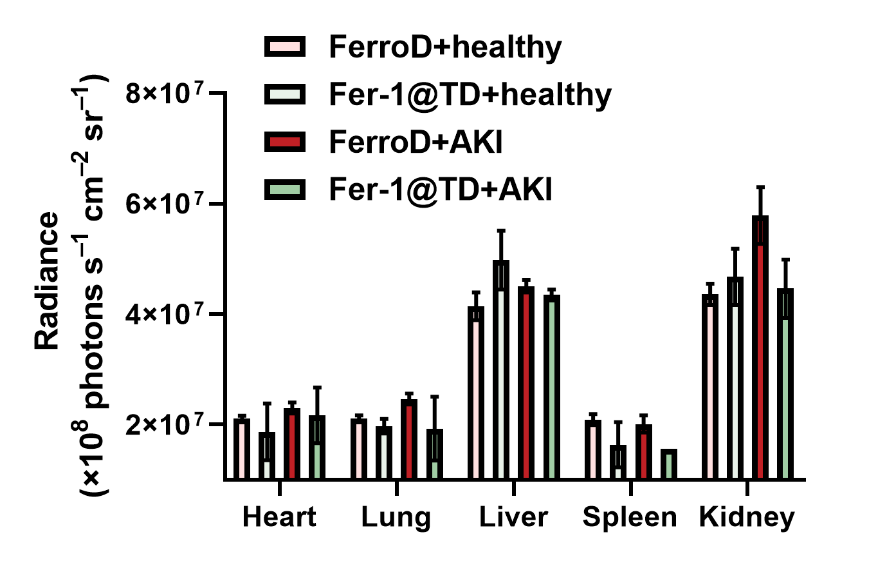


Figure S22 | The quantitation of the fluorescence intensity of main organs (heart, lung, liver, spleen, and kidney) of the normal and AKI mice at 1 h after intravenous injection of FerroD or Fer-1@TD.


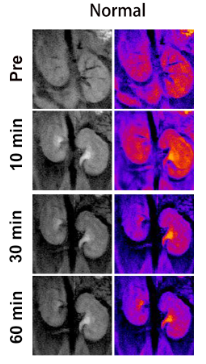


Figure S23 | The representative *T*_1_-weighted images at 10-, 30-, and 60-min post-injection of the Art-Gd probe in normal mice were acquired.


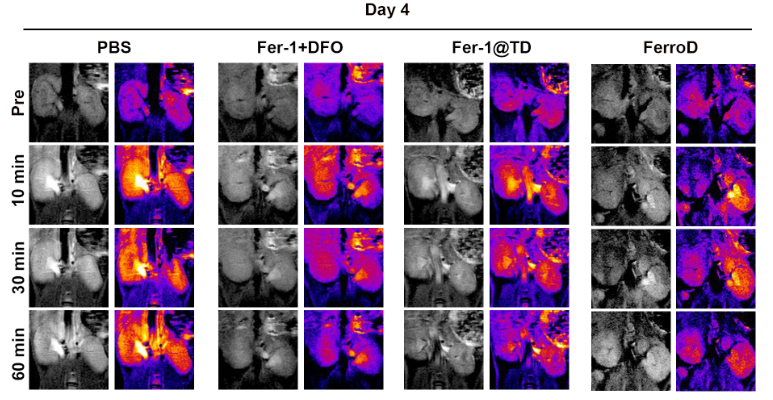


Figure S24 | **The MR images of evaluation the therapeutic efficacy in CDDP-induced AKI mouse models on Day 4 using Art-Gd probe.** The representative *T*_1_-weighted images at 10-, 30-, and 60-min post-injection of the Art-Gd probe in different groups, including PBS, Fer-1 + DFO, Fer-1@TD, and FerroD were acquired.


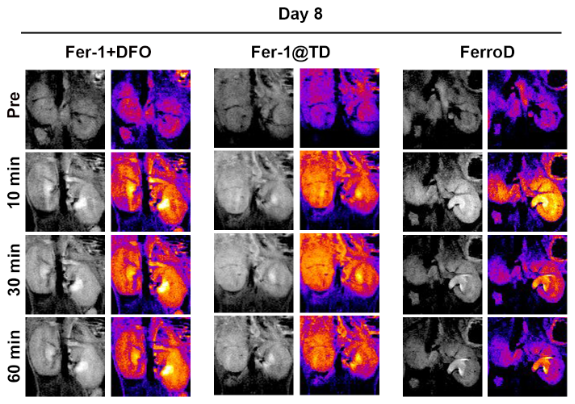


Figure S25 | **The MR images of evaluation of the therapeutic efficacy in CDDP-induced AKI mouse models on day 8 using Art-Gd probe.** The representative *T*_1_-weighted images at 10-, 30-, and 60-min post-injection of the Art-Gd probe in different groups, including Fer-1 + DFO, Fer-1@TD, and FerroD were acquired.


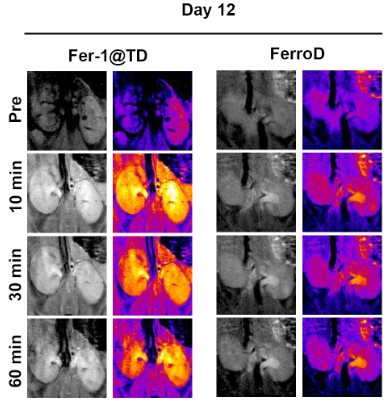


Figure S26 | **The MR images of evaluation of the therapeutic efficacy in CDDP-induced AKI mouse models on day 12 using Art-Gd probe.** The representative *T*_1_-weighted images at 10-, 30-, and 60-min post-injection of the Art-Gd probe in different groups, including Fer-1@TD and FerroD were acquired.


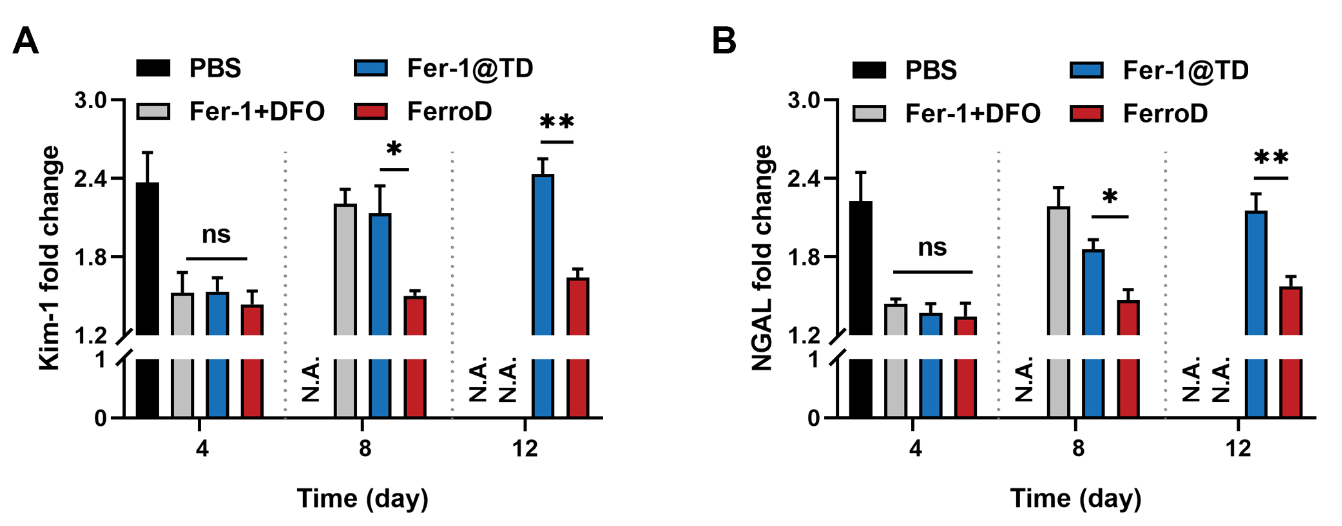
Figure S27 | Detection of the kidney function with Kim-1 and NGAL assays. (A, B) The fold-changes of the Kim-1 and NGAL in living mice (n = 3-4) after different treatments at different time points of days 4, 8, and 12. The ns represents no significance. **P* < 0.05, ***P* < 0.01 and ****P* < 0.001.


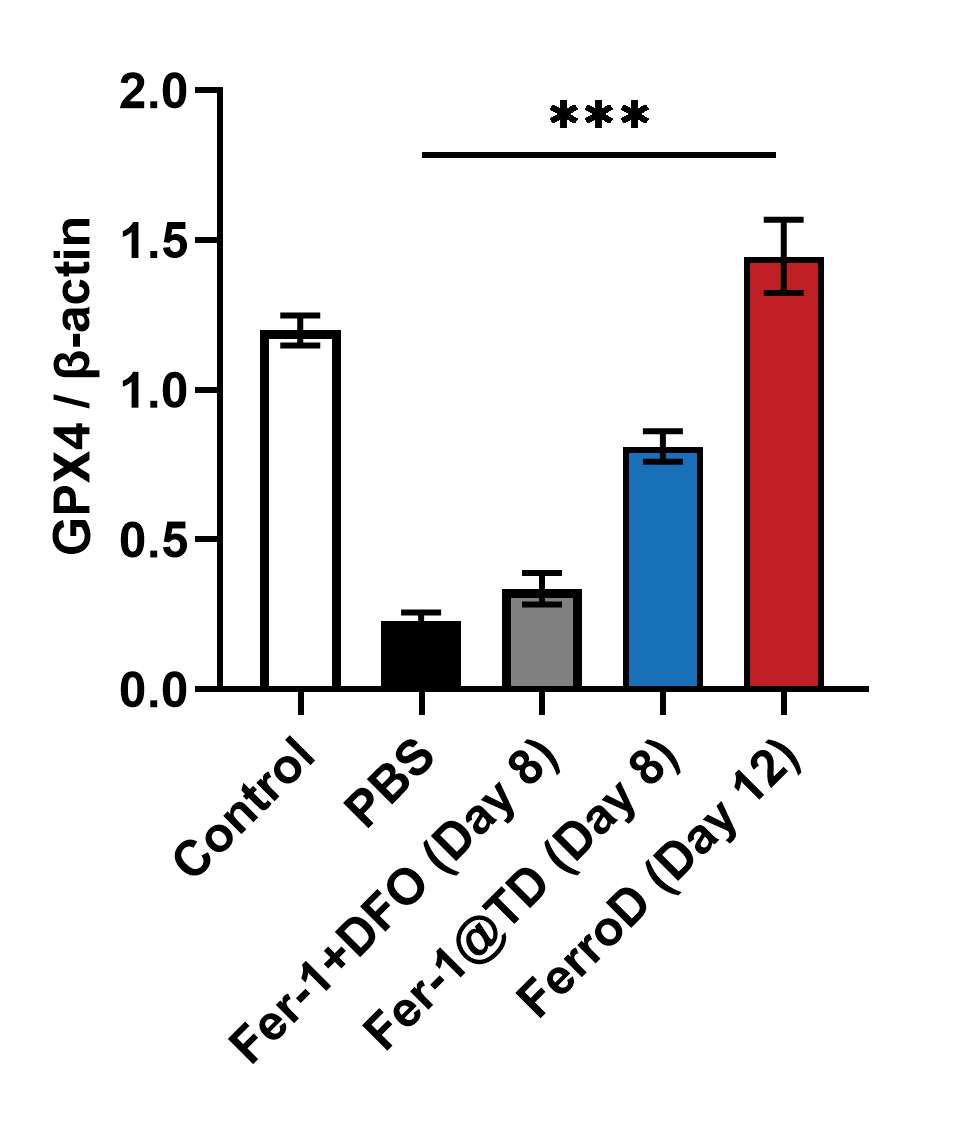


**Figure S28** | The semi-quantitative analysis of GPX4 levels in kidney tissues of CDDP-induced AKI mice after different treatments. ****P* < 0.001.


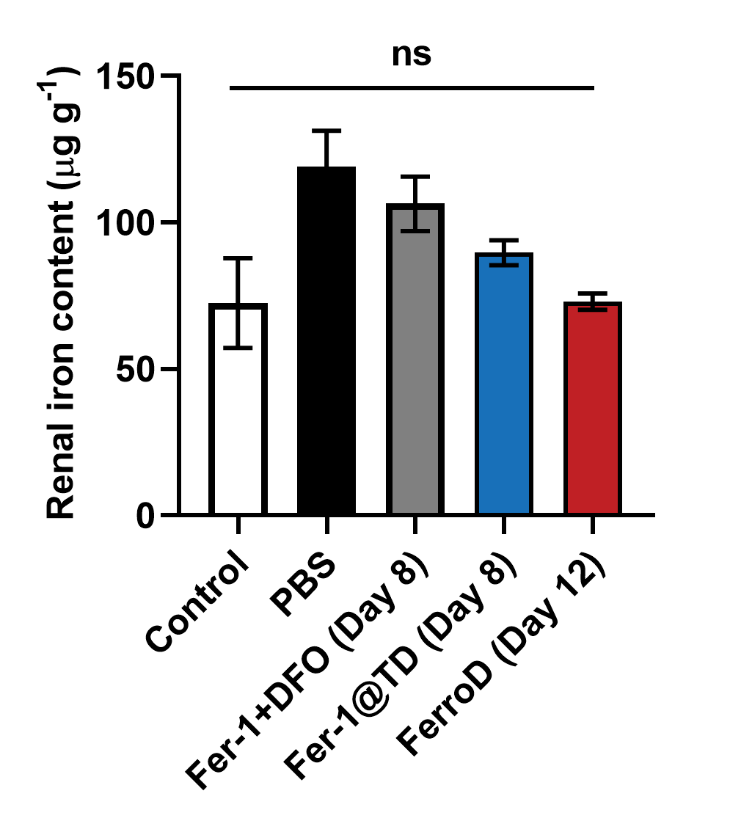


**Figure S29** | The total renal iron content of the mice with different treatments.
